# Human iPSC-derived midbrain organoids functionally integrate into striatum circuits and restore motor function in a mouse model of Parkinson's disease

**DOI:** 10.7150/thno.80271

**Published:** 2023-04-29

**Authors:** Xin Zheng, Deqiang Han, Weihua Liu, Xueyao Wang, Na Pan, Yuping Wang, Zhiguo Chen

**Affiliations:** 1 Cell Therapy Center, Beijing Institute of Geriatrics, Xuanwu Hospital Capital Medical University, National Clinical Research Center for Geriatric Diseases, and Key Laboratory of Neurodegenerative Diseases, Ministry of Education, Beijing 100053, China.; 2 Center of Neural Injury and Repair, Beijing Institute for Brain Disorders, Beijing, 100069, China.; 3 Center of Parkinson's Disease, Beijing Institute for Brain Disorders, Beijing, 100069, China.; 4 The Department of Neurology, Xuanwu Hospital Capital Medical University, Beijing 100053, China.

**Keywords:** Midbrain organoids, Human induced pluripotent stem cells, Parkinson's disease, Transplantation, Cell therapy

## Abstract

**Rationale:** Parkinson's disease (PD) is a prevalent neurodegenerative disorder that is characterized by degeneration of dopaminergic neurons (DA) at the substantia nigra pas compacta (SNpc). Cell therapy has been proposed as a potential treatment option for PD, with the aim of replenishing the lost DA neurons and restoring motor function. Fetal ventral mesencephalon tissues (fVM) and stem cell-derived DA precursors cultured in 2-dimentional (2-D) culture conditions have shown promising therapeutic outcomes in animal models and clinical trials. Recently, human induced pluripotent stem cells (hiPSC)-derived human midbrain organoids (hMOs) cultured in 3-dimentional (3-D) culture conditions have emerged as a novel source of graft that combines the strengths of fVM tissues and 2-D DA cells.

**Methods:** 3-D hMOs were induced from three distinct hiPSC lines. hMOs at various stages of differentiation were transplanted as tissue pieces into the striatum of naïve immunodeficient mouse brains, with the aim of identifying the most suitable stage of hMOs for cellular therapy. The hMOs at Day 15 were determined to be the most appropriate stage and were transplanted into a PD mouse model to assess cell survival, differentiation, and axonal innervation *in vivo*. Behavioral tests were conducted to evaluate functional restoration following hMO treatment and to compare the therapeutic effects between 2-D and 3-D cultures. Rabies virus were introduced to identify the host presynaptic input onto the transplanted cells.

**Results:** hMOs showed a relatively homogeneous cell composition, mostly consisting of dopaminergic cells of midbrain lineage. Analysis conducted 12 weeks post-transplantation of day 15 hMOs revealed that 14.11% of the engrafted cells expressed TH+ and over 90% of these cells were co-labeled with GIRK2+, indicating the survival and maturation of A9 mDA neurons in the striatum of PD mice. Transplantation of hMOs led to a reversal of motor function and establishment of bidirectional connections with natural brain target regions, without any incidence of tumor formation or graft overgrowth.

**Conclusion:** The findings of this study highlight the potential of hMOs as safe and efficacious donor graft sources for cell therapy to treat PD.

## Introduction

Parkinson's disease (PD) is a common neurodegenerative disease characterized by the degeneration of dopaminergic (DA) neurons in the substantia nigra pas compacta (SNpc) 1-3. The current standard clinical treatment for PD consists of levodopa administration and/or surgical deep brain stimulation 4. However, these treatments only temporarily alleviate symptoms and do not affect the underlying pathological progression 5. Cell therapy represents a promising new approach for the treatment of PD 6-10, and involves replenishing DA secretion from either allogeneic fetal tissues or autologous stem cell-derived DA cells to partially restore the loss of DA neurons and damaged nigrostriatal pathways 11-15. Animal studies and clinical trials using fetal ventral mesencephalon (fVM) tissues have shown that grafted DA neurons can survive, integrate and release DA into the host striatum, and can thereby alleviate Parkinsonian symptoms 16-22. Despite these promising results, fVM tissue transplantation failed to achieve the primary endpoints in two independent double-blind clinical trials. Moreover, it was found to be associated with certain graft-induced dyskinesias, possibly because of the complex graft composition and/or the negative impact of immune recognition on allogeneic grafts 23-25. Furthermore, ethical and logistical issues have impeded the widespread adoption of this approach in clinical practice 26.

The ground-breaking discovery of human induced pluripotent stem cells (hiPSCs) offers a new source of grafts for PD therapy 13, 27-29, which may be used as autologous 8, 11, 30-32 or allogeneic donor cells 29, 31, 33-35. Other cellular sources include embryonic stem cells (ESCs) and induced neural stem cells (iNSCs) 12, 36-39. Specific differentiation of iPSCs, ESCs, and iNSCs into DA precursors and engraftment into animal models of PD have shown efficacy in improving motor functions 36, 39, 40. The directed differentiation of two-dimensional (2-D) culture requires the use of soluble factors/morphogens to recapitulate the key biochemical pathways involved in the DA lineage specification that naturally occur during embryogenesis 29, 38, 41-51. Nevertheless, these 2-D culture systems are presently not at the level of sophistication and precision that is required to fully model the temporal and spatial regulation of DA neuron specification during three-dimensional (3-D) embryogenesis. In addition, soluble factors alone may not be sufficient to recapitulate the complicated components of a 3-D niche, such as cell-cell interactions and the extracellular matrix. Indeed, studies have shown that iPSC-derived 2-D DA precursors cells are sub-optimal when compared to fVM tissues with respect to the ability to restore drug-induced rotational behavior 52. Moreover, a higher number of surviving DA neurons in the striatum is required to reach a similar level of restoration after fVM transplantation 44. These results suggest that DA precursors derived through a 2-D culture may not be intrinsically identical to those obtained from normal embryonic midbrain.

Recent decades have witnessed technological advances that can induce pluripotent stem cells (iPSCs and ESCs) into various 3-D organoids, such as colon 53, 54, liver 55, 56, retina 57, and brain organoids 58, 59. To some extent, these organoids can model normal 3-D organ development and can be employed for mechanistic studies and drug screening 60. Jo et al. have successfully generated human induced pluripotent stem cell-derived 3-D midbrain organoids (hMOs) 61. However, whether hMOs can be used as donor grafts to treat PD has not been well studied. Compared with conventional 2-D culture, hMOs derived from iPSCs/ESCs are formed under complicated spatial and temporal regulations, to a certain degree reminiscent of human embryogenesis, which might render the organoids more compatible with the host for cell transplant therapy 62-64. Furthermore, the dynamic processes can provide stage-appropriate niche signals to induce a homogeneous population and tissue-specific cell types with more intact and mature functional activities 60. In the present study, we generated organoids from hiPSCs and transplanted them into the striatum of 6-hydroxydopamine (6-OHDA)-lesioned immunodeficient mice to investigate the safety and efficacy of the novel graft.

## Results

### Characterization of hiPSC-derived hMOs *in vitro*

**Figure 1:**
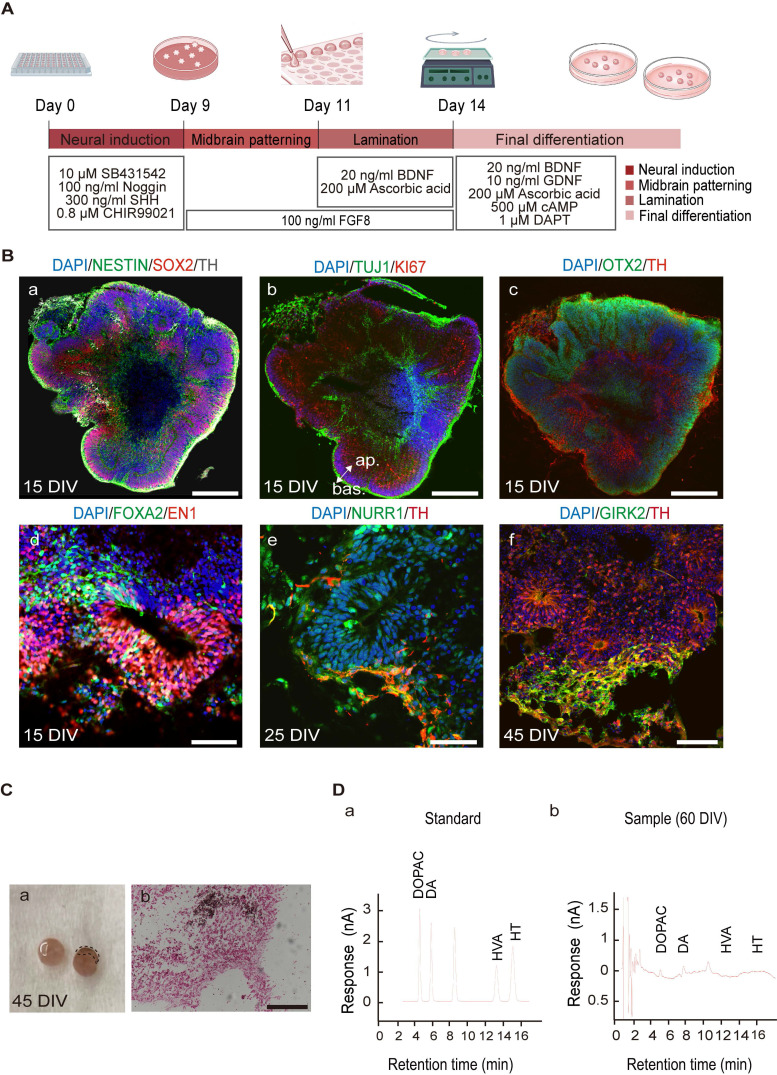
** Differentiation of human induced pluripotent stem cells (hiPSCs) to hMOs. (A)** Schematic representation of the procedure for differentiation of hiPSCs to hMOs. **(B)** Immunofluorescent staining for characterization of hiPSC-derived hMOs (*n = 10*). Scale bars: a, b, c, 250 μm; d, 75 μm; e, 50 μm; f, 100 μm. **(C)** Assessment of neuromelanin production in hMOs (*n = 6*). (a) A representative differential interface contrast (DIC) image of organoids with neuromelanin granule (within dotted box). (b) Fontana-Masson staining. Scale bar, 100 μm. **(D)** HPLC analysis of the whole organoids. The concentration of dopamine and the metabolite, 3,4-dihydroxyphenylacetic acid, were assessed following high potassium stimulation at 60 DIV (*n = 10*). FGF8, fibroblast growth factor 8; SHH, Sonic hedgehog; BDNF, brain-derived neurotrophic factor; GDNF, glial cell-derived neurotrophic factor; cAMP, dibutyryladenosine 3′,5′-cyclic monophosphate sodium salt; DAPT, 1-dimethylethyl ester; SOX2, sry-box transcription factor 2; TH, tyrosine hydroxylase; TUJ1, tubulin beta 3 class III; OTX2, orthodenticle homeobox 2; FOXA2, forkhead box A2; EN1, engrail-1; NURR1, nuclear receptor subfamily 4; GIRK2, G-protein-coupled inward rectifier potassium; DIV, Days *in vitro*; ap., apical; bas., basal; min, minutes; DA, dopamine; DOPAC, 3,4-dihydroxyphenylacetic acid; HVA, homo-vanillic acid; HT, 5-hydroxytryptamine.

hMOs were generated using a previously published method with minor modifications 61, 65 (Figure 1A). The hMOs obtained at different time points were analyzed by immunofluorescence staining and RT-PCR (Figure 1B, Figure S2). At 15 days *in vitro* (DIV), hMOs stained positive for the neural stem/progenitor cell markers NESTIN and SOX2, pan-neuronal and proliferative markers TUJ1 and KI67, embryonic midbrain progenitor marker OTX2, and floor-plate DA progenitor markers FOXA2 and EN1, indicating that the organoids were largely composed of midbrain DA lineage cells in the early stages of development (Figure 1B, a-d). At 25 DIV, hMOs stained positive for the midbrain post-mitotic DA cell markers, NURR1, and tyrosine hydroxylase (TH) (Figure 1B, e). At 45 DIV, expression of the midbrain A9 region-specific marker GIRK2 was confirmed (Figure 1B, f). In addition, the neuroepithelia within the organoids exhibited apical-basal polarity at 15 DIV, which could be explained by the expression of KI67, a proliferation marker mainly expressed on the apical side of organoids, and TUJ1+, a marker mainly expressed on the basal side (Figure 1B, b).

After characterizing the marker expression profile of specific markers, we assessed the functionality of hMOs. A neuromelanin-like structure started to appear at 45 DIV, and Fontana-Masson staining was performed to visualize neuromelanin, which is a black/brown-colored granular pigment that is normally produced by DA neurons located in the SNpc region 66. As shown in Figure 1C, the positively stained deposits were mainly located within the neuronal cytosol. To confirm the release of the neurotransmitter DA, we performed high-performance liquid chromatography (HPLC) analysis of the culture supernatant following high potassium stimulation and detected an abundant release of dihydroxyphenylacetic acid (3.806 ng/mL) and its metabolite dihydroxyphenylalanine (6.149 ng/mL) at 60 DIV (Figure 1 D, a, b). Secretion of the neurotransmitter serotonin was not detected in the cultures (Figure 1, D).

**Figure 2:**
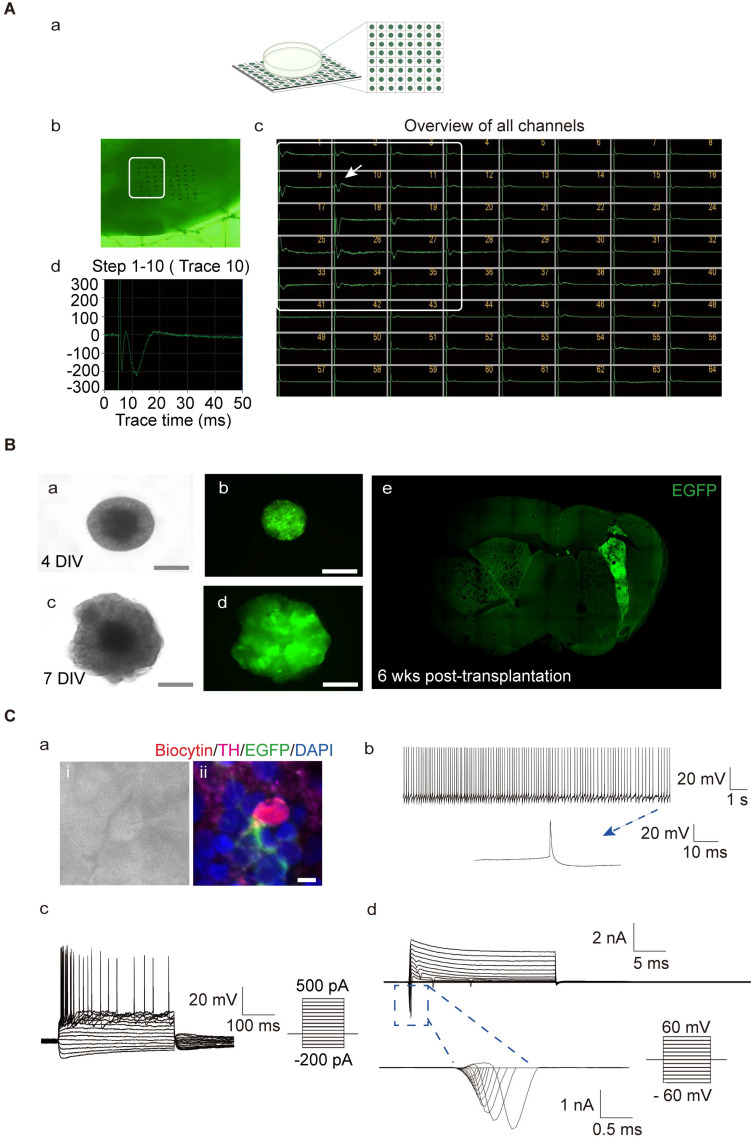
** Evaluation of the functional maturity of hMOs. (A)** The electrophysiological activity in whole organoids was assessed using a MED64 multi-electrode array system (*n = 4*). (a) Schematic representation of the electrophysiological signal processing system. (b) Photomicrographs of an organoid on a dish with the 64-electrode array. (c) Bursts occurring at the same time in different channels represented a network burst (the boxed region). (d) A representative spike cluster (arrow in c). **(B)** hiPSC- enhanced green fluorescent protein (EGFP) -derived hMOs were engrafted into the brain of severe combined immunodeficient (SCID) mice for examination of electrophysiological properties. (a-d) Microscopic images showing EGFP+ organoids on days 4 and day 7 (a, c, DIC images; b, d, fluorescence microscopic images). Scale bars, 2 mm. (e) A brain slice containing EGFP+ organoids 6 weeks post-transplantation. **(C)** Whole-cell current clamp recordings (*n = 3*). (a) Phase contrast image of a patched neuron and corresponding triple staining of the recorded neuron for EGFP, biocytin, and TH. (b) Regular spontaneous AP activity of a transplanted EGFP-cell. (c) APs evoked by step-current injections. (d) Representative voltage-dependent Na^+^ and K^+^ currents patched on a grafted neuron. Respective traces of inward Na^+^ in the blue box were expanded. Scale bars, 25 μm. DIV, days *in vitro*; wks, weeks.

To further understand the functional activity of the neuronal network formed in the hMOs, we utilized an MED64 multi-electrode array system, which is a non-invasive method to record extracellular field potentials generated by the whole organoid 57, 67, 68. In the naïve mouse group, field potential signals were captured in all channels, while, no signal was detected in PD mice without transplantation (Figure S1. A). Following transplantation, the signals were recovered (Figure 2A). We identified several synchronous network-wide bursts in the organoids, suggesting that neurons in hMOs developed appropriate network connections and electrophysiological activity (Figure 2A). To examine the electrophysiological properties of individual DA neurons, whole-cell patch-clamp recordings in acute brain slices of the engrafted hMOs were performed.

We first generated a reporter hiPSC line with lentiviral expression of enhanced green fluorescent protein (EGFP). EGFP was constitutively expressed during the process of differentiation of hiPSCs to hMOs (Figure 2B, a-d). EGFP+ organoids were transplanted into the brains of severe combined immunodeficient (SCID) mice. Six weeks post-transplantation, EGFP+ grafts were detected in all of the grafted mice, and the electrophysiological properties of the grafted cells were examined by whole-cell patch-clamp recording (*n = 3*; Figure 2B, e). The patched cells were confirmed to be positive for TH, EGFP, and biocytin (Figure 2C, a; Figure S1, B- and C- a). Whole-cell current-clamp recordings showed regular spontaneous action potentials (APs) in a transplanted EGFP+ cell with a long duration of single AP activity (>10 ms; Figure 2C, b; Figure S1, B- and C- b). Under current clamp mode, APs could be evoked by injection of currents from -200 pA to 500 pA in 20-pA incremental steps, and presented with a typical afterhyperpolarization 'sag' in the beginning of the responses and a delayed inward rectification at the end of the responses (Figure 2C, c; Figure S1, B- and C- c). In the voltage clamp mode, voltage-gated potassium (K^+^) channels and sodium (Na^+^) channels could be evoked from a holding potential of -60 mV to +60 mV in 10-mV steps (Figure 2C, d; Figure S1, B- and C- d).

### The optimal stage of DA differentiation for transplantation

**Figure 3:**
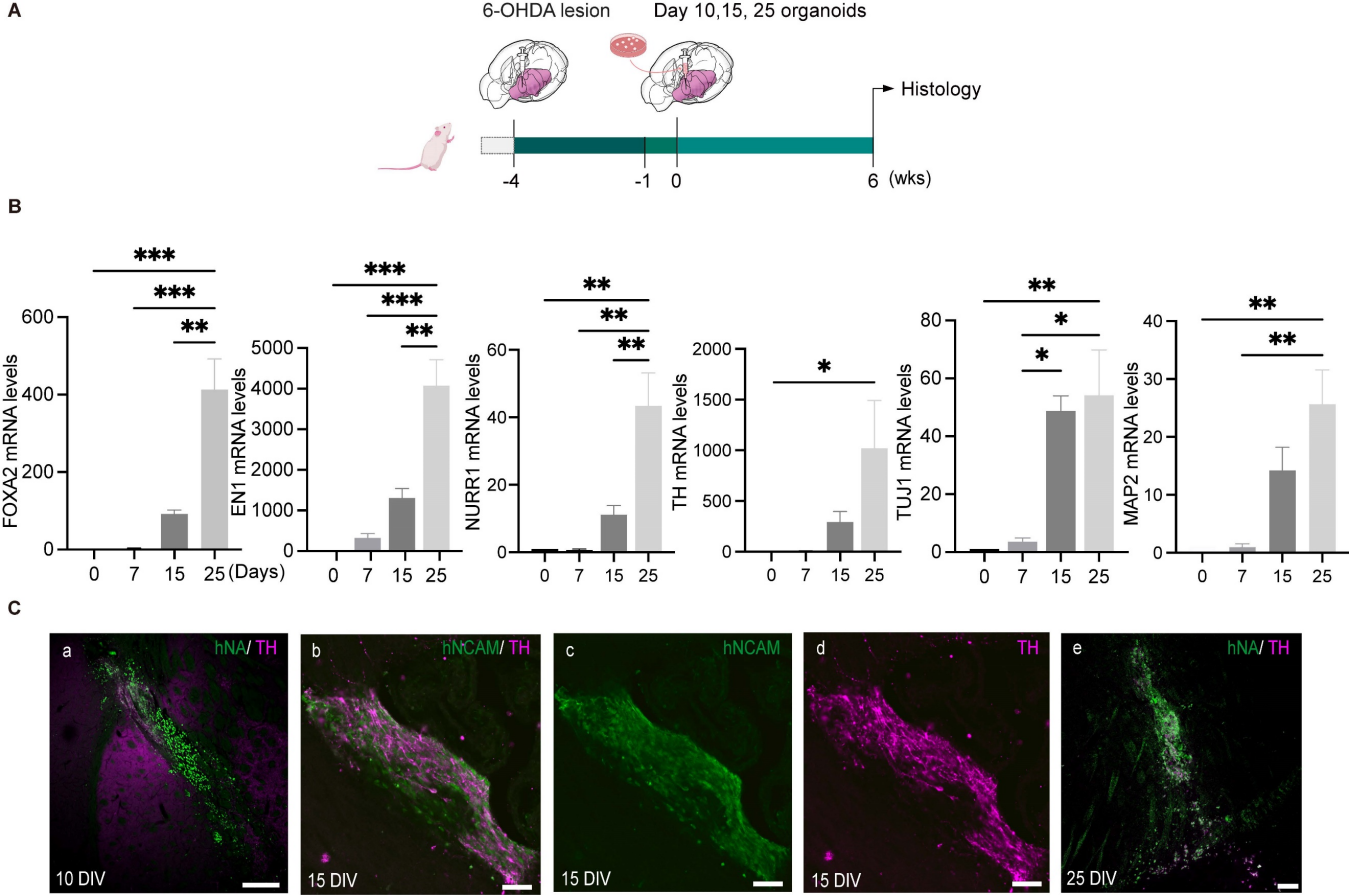
** The optimal differentiation stage of hMOs for transplantation. (A)** Schematic representation of the experimental design. **(B)** Representative RT-PCR results at different differentiation time points (*n = 6*). Results are expressed as fold changes compared to undifferentiated hiPSCs at day 0. Three independent experiments were conducted. **(C)** Survival and differentiation of hMOs following transplantation into brains of SCID mice (*n = 9*). Transplantation of day 10 (a), day 15 (b - d) and day 25 organoids. (e) Most organoids at 25 DIV died following transplantation, D 0, Day 0; DIV, Days *in vitro*. Scale bars: a, 250 μm; b, c, d, 50 μm; e, 100 μm.

An ideal stage of differentiation would ensure a good graft survival as well as committed specification into mature DA neurons following transplantation. To determine the optimal time window for differentiation, we first measured the mRNA expression of genes related to midbrain DA neuron development, including *FOXA2, EN1, NURR1, TH, TUJ1* and *MAP2*, in the organoids during the course of differentiation. Three different cell lines (hiPSCs-SB, hiPSCs-HW, hiPSCs-PJC) were used for evaluation. The expression of transcription factors associated with early DA lineage development, *FOXA2*, and *EN1* increased on day 7. In contrast, transcription factors expressed by post-mitotic DA cells, such as *NURR1* and *TH,* were detected on day 15 and peaked by day 25 (Figure 3B and Figure S4, A and B). The expression of the neuronal marker, *TUJ1* and* MAP2,* gradually increased during the period of time examined (days 0-25) (Figure 3B and Figure S4, A and B). Our previous study using iNSC-derived DA cells for transplantation showed that DA cells at a late mature stage cannot survive transplantation 36, 37. Therefore, we narrowed down the differentiation stage from day 10 to 25 for further engraftment tests. We transplanted organoids on days 10, 15 and 25 into the striatum of SCID mice (Figure 3C). Engrafted cells were identified using antibodies specific for human cell nuclei (hNA) or human neural cell adhesion molecules (hNCAM). After six weeks, the mice were sacrificed for analysis, and the surviving cells were detected in the brains of mice that were transplanted with day 10 and day 15 hMOs (Figure 3C, a to d). Most grafts with day 25 organoids died after transplantation (Figure 3C, e). In contrast, day 10 hMOs showed a good survival but only gave rise to a few TH+ cells (Figure 3C, a).

Compared to day 10 and day 25 hMOs, day 15 hMOs both survived and matured into DA neurons (Figure 3C, b-d). Based on these results, day 15 hMOs were selected for further investigation. On day 15, hMOs were characterized for expression of various markers and cell type composition prior to* in vivo* efficacy tests. The immunofluorescence staining results revealed that day 15 hMOs contained approximately 30.65% FOXA2-, 17.54% EN1- and 10.04% OTX2-positive cells, whereas a small portion of cells were positive for post-mitotic DA markers NURR1 (2.06%) and TH (0.79%) (Figure S2, B, a). On the other hand, day 25 hMOs were composed of 33.8% FOXA2, 21.4% EN1, 14% OTX2, 15.16% NURR1 and 11.6% TH-positive cells (Figure S2, B, b). Additionally, NURR1- and TH-positive cells increased to 50.44% and 32.22%, respectively, at 35 DIV (Figure S2, B, c).

Previous studies have shown that fVM tissues used in patient transplantation trials contain several non-DA neuronal populations, including GABAergic, glutamatergic, cholinergic and serotonergic neurons 69. Excessive numbers of serotonergic neurons in the grafts can lead to undesirable side effects, including graft-induced dyskinesia 70-72. Therefore, we assessed the composition of various cell types within the hMOs by staining for cell lineage markers, including DARPP32 for medial spiny neurons (MSNs), GAD67 for GABAergic, Vglut1 for glutamatergic, CHAT for cholinergic, and SEROTONIN for serotonergic neurons. None of the markers were detected in the immunofluorescence staining of hMOs (Figure S2, D, c, e, f). Since serotonergic cells in the fVM tissues have been reported to be associated with side effects, such as graft-induced dyskinesia 73, 74, in addition to immunofluorescent staining, we examined the mRNA expression of *NKX2.2, NKX6.1, HOXA2, HOXB, GBX2,* and *GATA3,* which are important transcription factors for the specification of serotonergic neurons 75-77. However, the expression of these genes was not observed (Figure S2, E). With regards to oligodendrocyte cell lineage, few Olig2+ cells were observed (< 1%) in day 15 organoids (Figure S2, D, b), and few O4+ cells were identified in day 35 organoids by immunofluorescence staining (Figure S2, D, a). In addition, mature MBP-positive oligodendrocytes (Figure S2, D, d) were not observed in day 15 hMOs.

### Effects of quercetin treatment on cultured undifferentiated cells and tumorigenicity study of transplanted organoids

**Figure 4:**
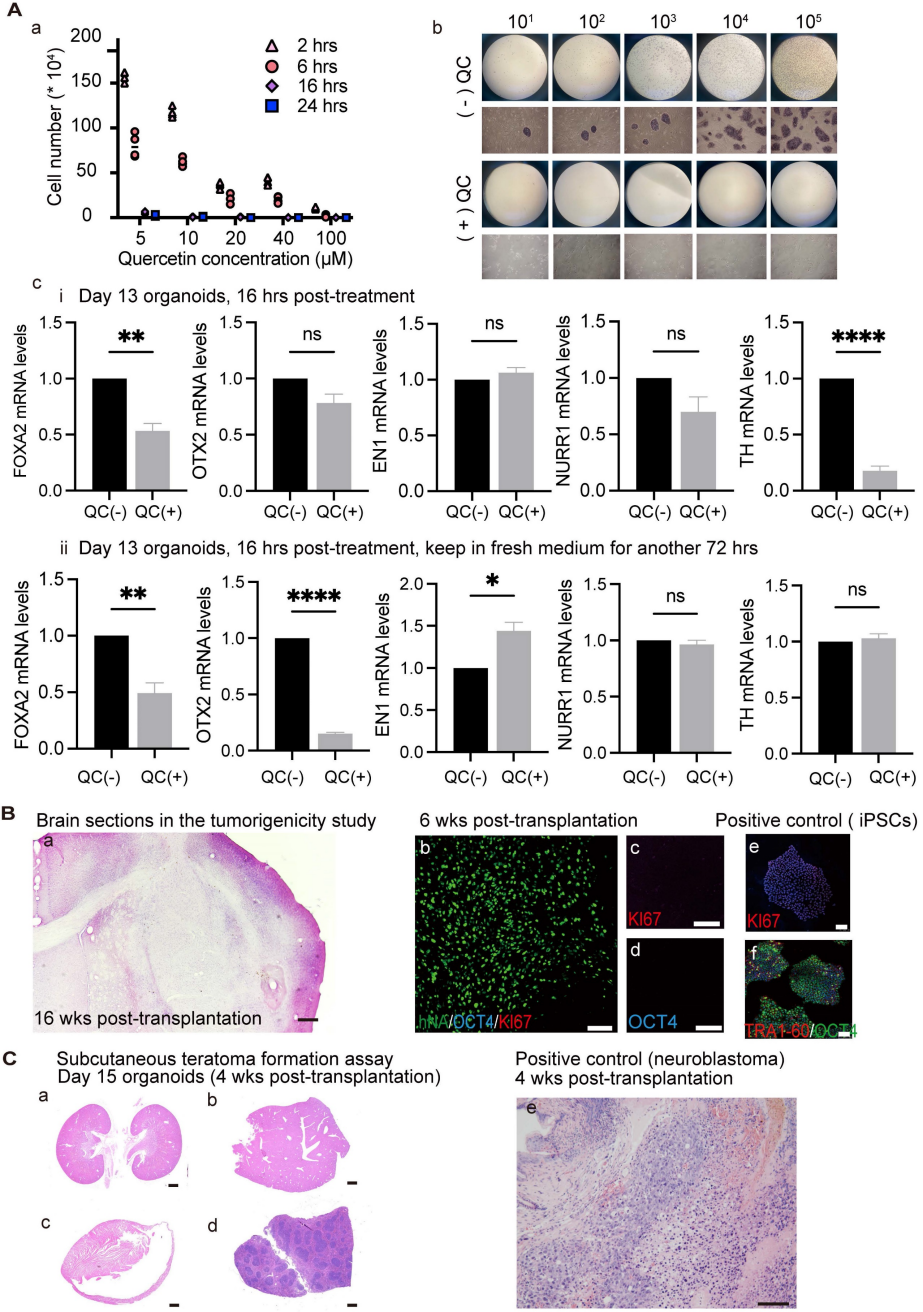
** Effects of quercetin (QC) treatment on cultured undifferentiated cells and tumorigenicity study of transplanted organoids. (A)** (a) Testing the optimal QC treatment conditions. Three independent experiments were conducted. (b) Response of different concentrations of hiPSCs to 40 μm QC. (*n = 3*) (c) Differentiation of organoids were evaluated after 40 μm QC treatment for 16 h. (i) Organoids were examined after the termination of QC treatment. (ii) Organoids were examined after having 3 days of recovery period.** (B)** Representative images of brain sections of the mice used in the tumorigenicity study (*n = 8*). H&E (a) / IF staining (b-d) of brain slices revealed no malignant or proliferative cells. Scale bars: a, 1mm; b-f, 100 μm. (e, f) Positive control for KI67 and OCT4 staining. **(C)** (a) Neither tumor formation nor tumor migration was detected in the subcutaneous space or in organs, such as the kidney (a), liver (b), heart (c), spleen (d). Scale bars: a-d, 1mm; e, 100 μm; (e) Representative images of grafts in the subcutaneous space of mice injected with neuroblastoma cell line as a positive control (*n = 6*). wks, weeks; hrs, h.

The most important concern for hiPSC-based cell therapy is to ensure safety by preventing the development of teratomas from contaminating pluripotent stem cells. A previous study showed that *BIRC5* (encoding survivin) is highly expressed in hiPSCs compared to differentiated cells 78. Treatment with the flavonoid quercetin (QC; 3,3',4',5,7-pentahy-droxyflavone), which targets a hPSC-specific antiapoptotic factor (i.e., survivin), leads to selective and efficient removal of pluripotent stem cells through apoptotic cell death. Importantly, this strategy did not interfere with DA neuron generation 11, 33. Because different cell lines exhibit different sensitivities to QC, we first searched for the appropriate dosage for the cells used in the present study. We treated 100,000 undifferentiated hiPSCs with 5, 10, 20, 40, and 100 μM quercetin (QC) for 2, 6, 16, and 24 h. After washing with fresh media, cells were further cultured for a total of 48 h and cell viability was measured using Trypan blue exclusion. Undifferentiated hiPSCs could be removed with an efficiency of higher than 99.99% when treated with more than 40 μM QC for more than 16 h, or more than 20 μM QC for more than 24 h (Figure 4A, a). To test the possibility that different concentrations of hiPSCs may have different drug sensitivities to a fixed drug concentration (40 μM QC for more than 16 h), undifferentiated hiPSCs were serially diluted by consecutive factors of 10 (10^5^, 10^4^, 10^3^, 10^2^, 10^1^, 10^0^) and seeded among human fibroblasts (100,000 cells) to identify residual hiPSCs using the AP-staining method (a surrogate marker for undifferentiated cells). The results verified that the QC treatment had higher than 99.99% efficiency in removing hiPSCs as no AP-positive colonies were found following the treatment (Figure 4A, b). Because survivin is an important marker in neuronal precursors, it is important to test whether QC affects the survival of DA neurons within organoids. We treated D13 organoids (mostly neuronal precursors) with 40 μM QC for 16 h and examined the effect on D14 organoids. The mRNA levels of DA lineage-related factors were affected by QC treatment (Figure 4A, c-i). It is possible that drug toxicity might have caused the above phenomenon; therefore, we removed QC from the culture, and assessed the organoids following a 3 day recovery period. At this time point, there were no significant differences in the mRNA levels of TH between QC-treated and -untreated organoids (Figure 4A, c-ii), suggesting that QC treatment may disturb immature neurons, but not irreversibly. Overall, our findings show that QC treatment lowers the number of undifferentiated cells, and can accordingly reduce the likelihood of tumor development following organoid transplantation.

Tumorigenicity was investigated at the same organoid stage as used in the transplantation studies. Hematoxylin-eosin (H&E) staining of 10-μm-thick brain slices revealed no proliferative or malignant cells (Figure 4B, a). The engrafted organoids were negative for OCT4 and KI67 at 6 weeks post-transplantation, indicating low tumorigenic risk (Figure 4B, b-d). Day 15 organoids were injected into the subcutaneous space of SCID mice along with Matrigel; this is considered the most sensitive method for detecting teratoma formation. Neither tumor formation nor migration was detected in the subcutaneous space or in organs, such as the kidney, liver, heart, and spleen (Figure 4C, a-d). As a positive control, 600,000 neuroblastoma cells were injected subcutaneously and tumors were detectable at approximately 4 weeks.

### Transplantation of hiPSC-derived hMOs improves the motor function of PD mice

**Figure 5:**
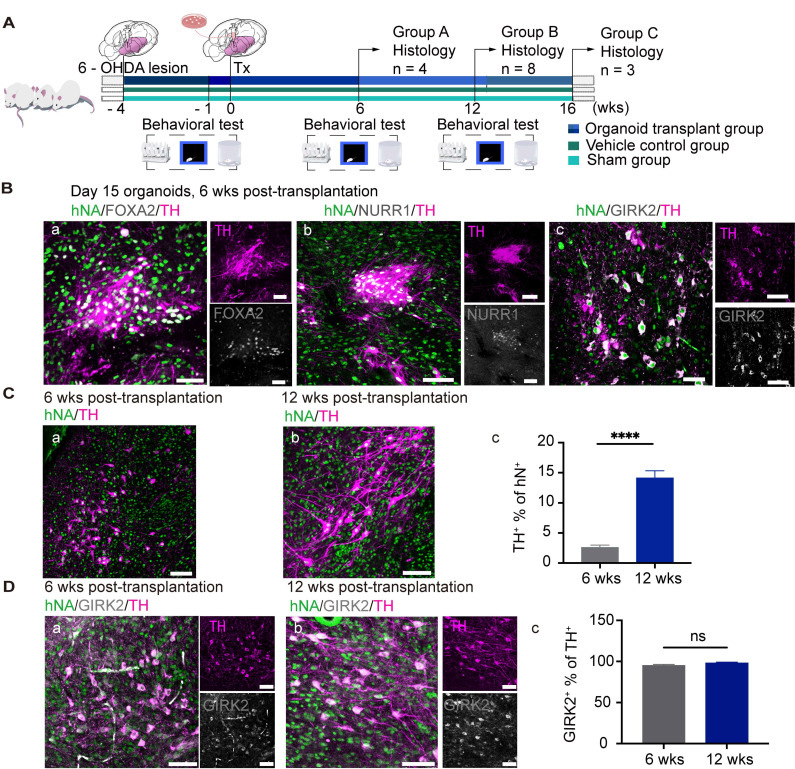
** Transplantation of hiPSC-derived hMOs into PD mice. (A)** Schematic representation of the experimental design and timeline of behavioral tests. Small subsets of mice in groups A and C were sacrificed for histology analysis at a specific time point, while a subset of mice in group B *(n = 8)* were examined for behavioral data collection. **(B)** Immunofluorescent staining confirmed the differentiation and maturation of engrafted organoids *in vivo (n = 8).* Scale bars, 50 μm. **(C)** (a-c) Representative confocal microscopic images at two time points following transplantation and the proportions of TH+ cells among the engrafted cells were plotted (2.57% for 6 weeks *[n = 4]* and 14.11% for 12 weeks *[n = 8]*). **(D)** Representative confocal microscopic images at two time points of transplantation and the proportions of GIRK2+ in TH+ graft areas (95% for 6 weeks *[n = 10]* and 98% for 12 weeks *[n = 20]*); *p < 0.05, ** p < 0.01, **** *p* < 0.0001 by t - test. Scale bars, 100 μm. Tx, transplantation; wks, weeks.

Next, we transplanted day 15 hMOs into the striatum of unilaterally 6-OHDA-lesioned SCID PD mice (Figure 5A). Mice were sacrificed for histological analysis at 6-, 12-, and 16- weeks post-transplantation. Engrafted cells were identified using hNA and hNCAM staining. The differentiation and maturation of engrafted organoids *in vivo* were confirmed by co-labeling with mDA-specific markers, including FOXA2, NURR1, and TH, and the A9 region-specific DA neuron marker GIRK2 (Figure 5B, a-c).

The TH+ grafts at 12 weeks post-transplantation presented a more mature morphology with a larger soma and more extensive arborization compared with those at 6 weeks (Figure 5C, a and b), suggesting that the grafts had gradually maturated over time. At 6 weeks post-transplantation, approximately 2.57% of the surviving hNA+ cells were co-labelled with TH (Figure 5C, c); At 12 weeks post-transplantation, the proportions of engrafted (hNA+) cells that co-expressed TH was 14.11% (Figure 5C, c). A9 DA neurons inside intrastriatal grafts are essential for the recovery of motor performance of PD mice.

An authentic A9 phenotype of the grafted cells was confirmed via co-labeling for TH and GIRK2 (specifically expressed by A9-subtype mDA) 65. In in *vivo* bioassays, 90% of GIRK2+ neurons were co-labeled by TH at 6- (Figure 5D, a) and 12- (Figure 5D, b) weeks following transplantation (Figure 5D, c), suggesting that the A9 mDA neurons had survived and matured in the striatum of the PD mice to rescue behavioral deficits. It is also important to characterize the cell identity of the engrafted cells which were TH-negative (Figure 5). The immunofluorescence staining results revealed a high expression of OTX2+ (74.83% of hNA+) and FOXA2+ (49.50% of hNA+) in the grafts following implantation (Figure S6, A, C, E). Almost all of OTX2+ or FOXA2+ cells were co-labeled with human neural cell marker hNCAM (Figure S6, B, D), suggesting that these cells were dopaminergic lineage cells but might not have maturated enough to generate tyrosine hydroxylase. In addition, we did not detect any grafted-derived astrocytes, oligodendrocytes, or other types of neurons (e.g., glutamatergic neurons) within the grafted deposit (Figure S6, F).

To examine the efficacy of grafts in restoring motor functions in PD mice, behavioral tests were performed, including apomorphine (APO)-induced rotation, open field, and rotarod tests. PD mice that received hMO grafts showed a statistically significant improvement in all the three tests, which was not observed in the vehicle control group (Figure S3, A and B). These results suggested that transplantation of hMOs can restored the motor functions damaged by 6-OHDA lesioning and that the pathophysiological changes related to the improvement of motor disorders may be associated with amelioration of the host microenvironment. We found that the mRNA expressions of proinflammatory cytokines, interleukin *(IL)-1β* and *IL-6*, which are involved in DA neurotoxicity, was down‐regulated by 7.85- and 36.66‐fold, respectively, in the striatum tissues of hMO-transplanted mice (p ≤ 0.01) compared with that in those of 6-OHDA-lesioned mice without engraftment (Figure S3, C). In addition, mRNA levels of* Nrf2* and *Hmox1*, which protect mDA neurons against inflammasome activation, were upregulated by 1.2- and 3.6-fold, respectively, in the striatum tissues of hMO-transplanted mice compared with that in those of the 6-OHDA-lesioned mice without engraftment (Figure S3, C). These findings suggest that organoid grafts could have contributed to formation of a regulatory niche that may balance proinflammatory and anti-inflammatory responses in 6-OHDA‐lesioned striatum.

### Engrafted neurons progressively send out axonal projections to innervate target brain regions

**Figure 6:**
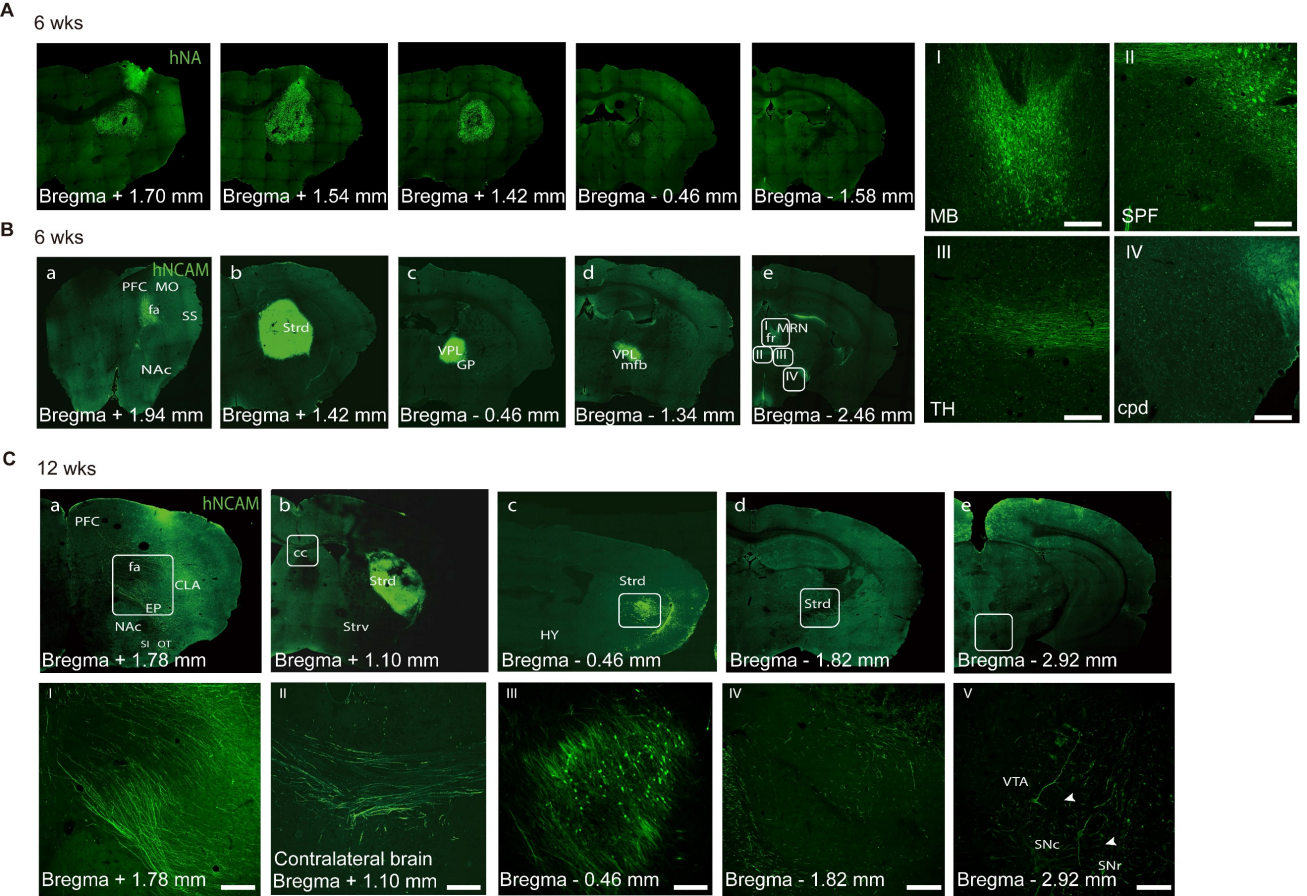
** Axonal projections of engrafted neurons. (A)** Representative immunofluorescence images revealing the distribution of transplanted cells at 6 weeks. **(B)** Immunofluorescent staining of hNCAM demonstrating graft-derived fibers innervating into host brain regions, including cerebral cortex (a), striatum (b), medial forebrain bundle (c, d), and midbrain (e). The boxed areas were magnified in I-IV. Scale bars, 250 μm. *(n = 4)* **(C)** Immunofluorescent staining of hNCAM demonstrating graft-derived fibers innervating into host brain regions at 12 weeks *(n = 8);* I-IV corresponds to a-e, respectively. Scale bars, 250 μm. wks, weeks; PFC, prefrontal cortex; fa/CC, corpus callosum; MO, somatomotor cortex; SS, somatosensory cortex; NAc, nucleus accumbens; Strd/ Strv, dorsolateral/ventral striatum; GP, globus pallidus; VPL, ventral posterolateral nucleus of the thalamus; mfb, medial forebrain bundle; HY, hypothalamus; TH, thalamus; MB, midbrain; MRN, midbrain reticular nucleus; fr, fasciculus retro-flexus; SPF, sub-parafascicular nucleus; CLA, claustrum; EP, endopiriform nucleus; SI, substantia innominate; OT, olfactory bulb; SNr/ SNc, reticular/ compact part of substantial nigra; VTA, ventral tegmental area.

Specific axonal projections and their integration into host neuronal circuits are critical for the functional recovery of mice with brain injury 79-81. To analyze the projections derived from the graft, we used human cell-specific hNCAM antibodies to label the engrafted cells and their projections. During the examination, the graft bolus mostly remained in the same position; however, the processes (dendrites and axons) extended far away from the graft core (Figure 6A). The brains collected at 6- and 12- week post-transplantation were coronally sliced and analyzed along the antero-posterior axis.

At six weeks, a few hNCAM+ fibers had extended along the corpus callosum from the striatally grafted region to the cerebral cortex (Figure 6B, a), mostly in the primary somatomotor cortex of the prefrontal cortex (PFC), which is a natural A9- or A10-neuron target region. No hNCAM+ fibers were observed in the primary somatosensory area. In contrast, a portion of hNCAM+ fibers had extended posteriorly and entered to the globus pallidus, ventral posterolateral nucleus of the thalamus and medial forebrain bundle (Figure 6B, c and d). Some hNACM+ fibers continued to outgrowth across the midbrain motor-related nucleus, such as the midbrain reticular nucleus and fasciculus retro-flexus, into the subparafascicular nucleus and posterior complex of the thalamus (Figure 6B, e: white box in B-e corresponding to the I, II, and III). A few hNCAM+ fibers eventually extended as far as the posterior cerebral peduncle (cpd) (Figure 6B, e: white box in B-e corresponding to the IV).

**Figure 7:**
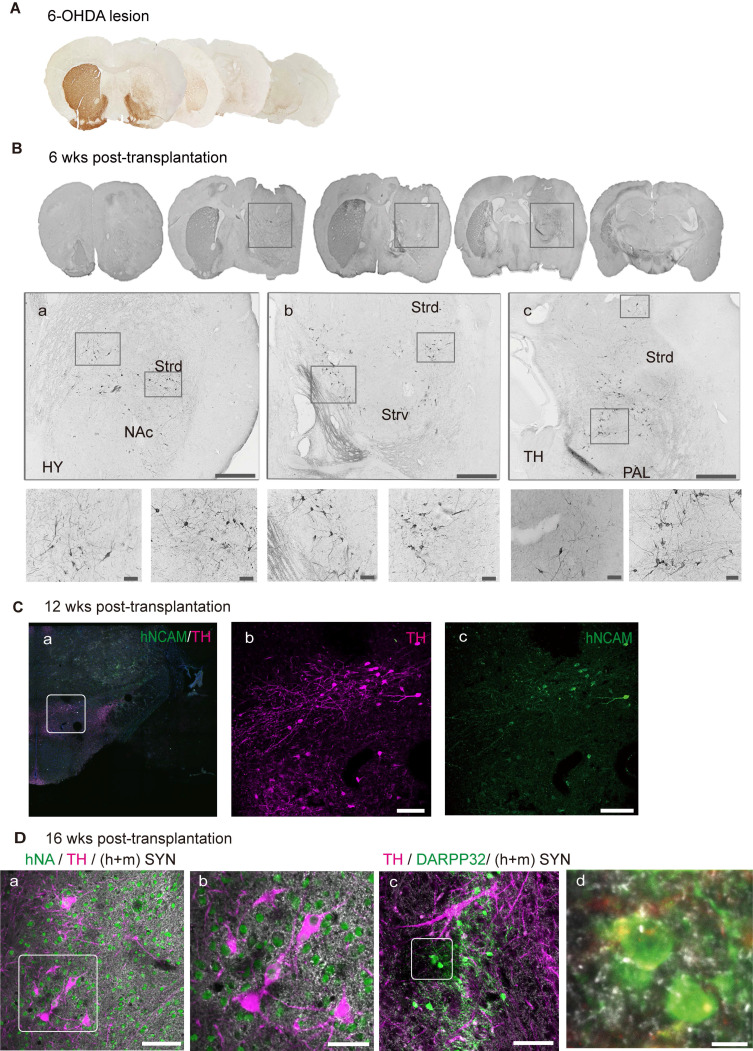
** Graft-derived DA neurons innervate into host neuronal circuitry. (A)** Immunohistochemical analysis revealed degeneration of unilateral endogenous TH-positive midbrain dopaminergic neurons in the substantia nigra (SN) and striatum in 6-OHDA-lesioned mice. **(B)** Graft-derived DA neurons in the lesioned hemisphere. Scale bars, 100 μm.** (C)** TH+/hNCAM+ fibers innervated into ipsilateral reticular/compact part of the SN. The boxed areas in a were magnified in b and c. Scale bars, 250 μm.** (D)** Transplanted DA neurons received synaptic (h+m Syn) inputs from host and/or grafted neurons (a, the box area was magnified in b). Grafted DA neurons extended the axons to adjacent medial spiny neurons (the boxed area in c was magnified in d). Scale bars, a, b, c, 50 μm; d, 7.5 μm; 6-OHDA, 6-hydroxydopamine; wks, weeks; NAc, nucleus accumbens; Strd/ Strv, dorsolateral/ventral striatum; TH, thalamus; PAL, pallidum.

After 12 weeks, prominent hNCAM+ projections were observed covering the claustrum and endopiriform nucleus, with a few fibers reaching as far as the rostral olfactory bulb and the substantia innominata (Figure 6C, a and I). Analysis of more rostral structures, including the dorsolateral/ventral striatum (Strd/Strv) and nucleus accumbens (NAc/ACB), revealed the absence of obvious graft-derived innervation into the Strv and NAc regions, mainly the A10 targets. The grafts were placed in the Strd (Figure 6C, b, c, and III), an area that has been confirmed to be strongly associated with improved motor function in cell therapy studies. A closer look at the direction of axonal extension revealed that several hNCAM fibers had travelled along the corpus callosum into the contralateral cortex (Figure 6C, II). We observed a considerable growth of the hNCAM+ terminal network in the Strd closer to the substantia nigra (SN) than that in mice at 6 weeks (Figure 6C, d, IV). A small portion of the fibers extended to the reticular/compact part of the SNr/SNc and ventral tegmental area (Figure 6C, e and V; Figure 7C, a-c).

### Graft-derived DA neurons innervate into host circuitry

Histological analysis revealed the degeneration of unilateral endogenous TH-positive midbrain dopaminergic neurons in the SN and Str in vehicle control mice, suggesting a complete Str lesion (Figure 7A), which was consistent with the results of APO-induced rotational asymmetry. To examine the outgrowth of DA neurons, we stained the sections for TH and found that most of the TH+ grafts were located at the grafted site within the dorsal striatum (Figure 7B). To identify the axonal extension of TH+-grafted neurons, we double-stained the sections for TH and hNCAM. At 12 weeks post-transplantation, a small portion of TH+/hNCAM+ fibers continued to extend and entered the ipsilateral SNr and SNc, as well as the ventral tegmental region (Figure 7C, a-c).

Functional recovery of the graft area is not only dependent on the survival of DA neurons and increased release of dopamine but also depends on graft integration into the host neuronal circuits to achieve long-term alleviation of PD-related motor symptoms 28. We used a synapsin antibody that can recognize both human and mouse species to identify the synapses formed, and staining revealed that graft-derived TH+ neurons received synaptic inputs from the host (mouse) and/or engrafted (human) neurons (Figure 7D, a and b). Additionally, midbrain DA neurons projected naturally onto MSNs. To examine whether the engrafted neurons had formed synapses, we stained for DARPP32, an MSN marker, together with TH and synapsin (Figure 7D, c and d; Figure S4, C). These results indicate that engrafted DA neurons may have formed synapses with endogenous MSNs.

### Retrograde tracing reveals axonal outgrowth patterns

**Figure 8:**
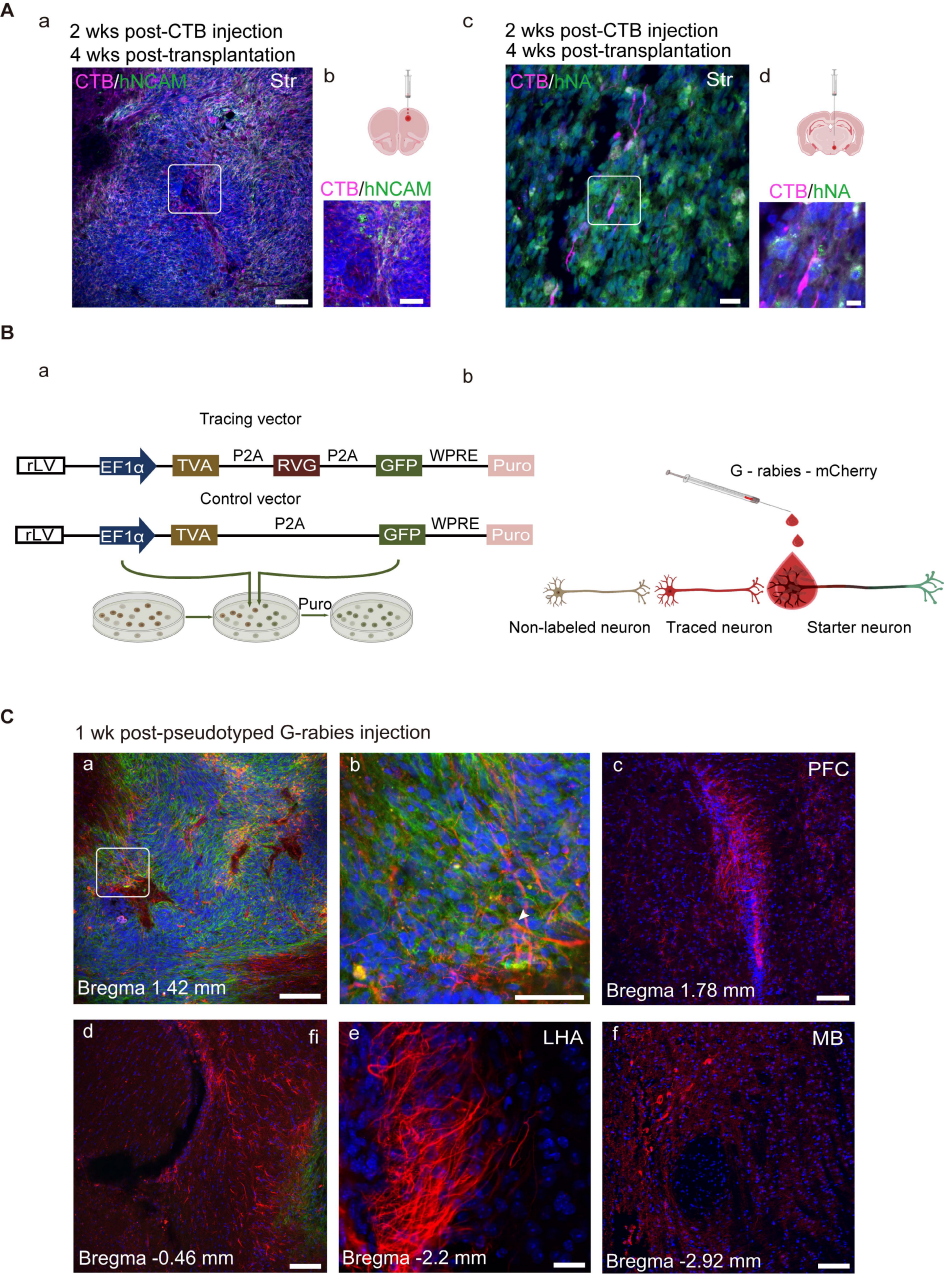
** Transplanted neurons connect with host neurons. (A)** Grafted neurons located in the striatum projecting to prefrontal cortex (PFC) and hypothalamus (HY) (the boxed areas in a and c were magnified in b and d, respectively). **(B)** (a) Ideograph illustrating the rabies virus tracing system. (b) Ideograph illustrating GFP+ starter neurons infected with G-rabies which then started expressing mCherry. Traced neurons were infected by retrogradely transmitted G-rabies and were labeled with mCherry. **(C)** Monosynaptic tracing identified the early host-to-graft connectivity *(n = 4)*. (a) Starter neurons were mainly located at the transplantation site and retrogradely infected adjacent grafted and/or host cells (arrows highlighted the starter neurons with GFP+/mCherry+). (b-f) Scattered traced neurons located at several brain regions, including PFC, fimbria, dorsolateral striatum, lateral hypothalamus area, and midbrain. Scale bars, a, c, d, e, f, 250 μm; b, 75 μm. Puro, puromycin; fi, fimbria; LHA, lateral hypothalamus area.

Cholera toxin subunit B (CTB), a retrograde axonal tracer, was microinjected into the rostral area PFC or posterior hypothalamus (HY) in separate groups of mice. CTB deposits within the intended target site were visualized using a CTB-specific antibody. To confirm that the CTB deposit was clearly separated from the graft itself, CTB and hNA were double-stained, and the results showed that grafted cells were absent at the CTB injection site (Figure S4, D). Two weeks later, the brain sections were double-stained with CTB and hNA or hNCAM, and CTB-labeled grafts were detected in the striatum (Figure 8A), suggesting that the engrafted cells had projected into the PFC and HY by 4 weeks post-transplantation.

The selective retrograde transmission of the rabies virus across a single synapse permits precise identification of the host presynaptic input onto the transplanted cells 82.

To achieve monosynaptic retrograde transmission, hiPSC-derived organoids expressing lentiviral vectors (a rabies helper construct) encoding TVA (the subgroup A avian sarcoma leukosis virus (TVA), rabies glycoprotein (RVG), and GFP were transplanted into the midbrain (Figure 8B, a; Figure S4, E). Histone-tagged GFP could be used for starter cell confirmation; the TVA receptor and RVG, upon primary infection with EnvA-pseudotyped rabies, could promote monosynaptic retrograde transmission of the rabies virus (Figure 8B, b). We employed a control vector, including GFP and TVA, but missing RVG in parallel to the control for non-specific labeling. Four weeks after transplantation, differentiated DA neurons co-labeled with GFP and TH were observed at the engraftment site (Figure S4, F). To mark the synaptic inputs onto the grafted cells (hereafter referred to as starter neurons), EnvA-pseudotyped G-rabies was injected into the same site as the hMO transplants (Figure 8C, a).

GFP expression was confirmed in starter cells. After infection with the G-rabies vector expressing mCherry, starter neurons expressing TVA receptors may be infected with G-rabies and easily recognized by the co-expression of GFP and mCherry. G-rabies virus can assemble into infectious particles in the starter cells because these neurons express RVG. Because the rabies viruses prefer to spread retrogradely through active synapses, every neuron that forms presynaptic connections with the starter neuron, known as a traced neuron, will be transduced with mCherry 83. Therefore, only red fluorescence (mCherry, but not GFP expression) can be identified in the traced neurons connected to the starter neurons. The mice were micro-injected with the pseudorabies virus and sacrificed for histological analysis after one week. We detected grafted neurons that were successfully infected with the pseudorabies virus and identified traced neurons located inside the graft as well as in several brain regions, including the PFC, fimbria, Strd, lateral HY area, and midbrain (Figure 8C, c-f).

## Discussion

In the present study, hiPSC-derived hMOs were transplanted into an immunodeficient mouse model of PD. These hMOs matured into DA neurons that expressed FOXA2, NURR1, TH, and GIRK2, and displayed electrophysiological activity, which resulted in improved motor function of PD mice. Additionally, the engrafted hMOs showed extensive neurite/axon extension and formed strong synaptic connections with host neurons.

Clinical trials using fVM tissues to treat PD have shown efficacy in some patients; however, this has also raised ethical, logistical, and heterogeneity concerns. hMOs obtained from hiPSCs could potentially solve the ethical and logistical issues associated with the use of embryonic fVM tissues. Moreover, hMOs may have an advantage in terms of cellular diversity, as fVM tissues are primarily composed of non-neuronal cells such as astrocytes, microglia, and endothelial cells, with neurons making up only about 5.6% of the total cell population. Within this small proportion of neurons, approximately 70% are of the DA lineage, 30% are GABAergic, and 2-3% are glutaminergic. 84, 85. Owing to the technical challenge of precisely isolating the ventral mesencephalic regions in small embryos aged 7-9 weeks post-conception, grafts obtained from this tissue often contain contaminating cells, including serotonergic neurons. These cells were associated with significant side effects, such as graft-induced dyskinesia, in two double-blind clinical trials. 86, 87. In the present study, hMOs derived from iPSCs on day 15 were mainly comprised of neuronal lineage cells, and no astrocytes or oligodendrocytes were detected (Figure S6, F). Over 30% of the neuronal cells were DA lineage cells on day 15 (with 30.64% FOXA2+, 17.54% EN1+, 10.04% OTX2+, 2.10% NURR1+ and 0.80% TH+; Figure S2, B, a), and no GABAergic, glutaminergic, or serotonergic neurons were detected (Figure S2, D). Additionally, the expression of genes associated with serotonergic cell specification during development was not detected (Figure S2, E). These results suggested that hMOs possessed a more homogeneous cellular nature than fVM tissues. Therefore, the non-DA cells in the graft may not give rise to obvious side effects when engrafted into a relatively small and less complex rodent brain. However, the human brain has a more complicated structure, composition, regional difference, and larger size, and hence the presence of non-DA cells is associated with a much higher degree of uncertainty with respect to clinical outcomes. The relatively more homogenous nature of hMOs might indicate a lower risk for future clinical translation.

hiPSC-derived DA precursors in 2-D culture can also address some of the above-mentioned issues associated with fVM tissues. However, the biggest concern with regard to hiPSC derivatives is the tumorigenic risk associated with a possibly incomplete differentiation of pluripotent stem cells 88-91, in spite of the effort that has been made to eliminate such contaminating cells 92-100. Compared with single-cell cultures, organoid culture relies on inner structural contact/support and intrinsic signals that orchestrate/synchronize the development of all involved cells at the organ/tissue level.

This process mimics embryogenesis, and the resultant organoid would, at least theoretically, have a low tumorigenic risk, comparable to that of fVM tissues. In the present study, no tumor formation or graft overgrowth was observed following transplantation into the brain of immunodeficient mice, and the grafts were negative for KI67 and OCT4 (Figure 4B), confirming low tumorigenic risk.

Some studies have reported that 2-D DA cells derived from iPSCs may not be equivalent to DA cells in fVM tissues 101. A greater number of iPSC-derived 2-D DA cells would be required to achieve a similar level of restoration of motor functions compared to fVM transplantation. About 500 - 700 surviving fVM DA neurons are sufficient to reverse the drug-induced rotation 52, 102. whereas thousands of iPSC-derived 2-D DA neurons are required to fully reverse drug-induced rotation 38, 39, 88, 93.

In the present study, the same number of 2-D-derived DA neurons were transplanted to compare the therapeutic effect between 2-D and 3-D cultures. No statistically significant difference was observed in movement improvement between the two different culture conditions at 6- and 12- weeks following transplantation (Figure S5, D, a-b). Interestingly, the functional recovery of motor function started to be observed at 6 weeks post grafting in the group receiving 3-D culture (n = 6), whereas in the group receiving 2-D culture, such recovery only began to be detected at 12 weeks (Figure S5, D, c-d), suggesting that organoid graft may assume different temporal kinetics following transplantation than 2-D cells. The higher efficiency of fVM DA cells might be related to their greater capacity for arborization. Viable fVM DA neurons can extend processes up to 6 mm and arborize almost the whole striatum 52; In contrast, iPSC-derived 2-D DA neurons extend processes up to 2-3 mm and arborize only approximately 10% of the striatum 103. In the present study, hMOs gave rise to approximately 200-800 of surviving DA neurons and reversed apomorphine-induced rotation by 6 weeks post-transplantation (Figure 5D). Further studies are warranted to investigate whether a smaller number of hMO-derived DA neurons is sufficient to restore the motor function. Furthermore, hMO-derived DA neurons not only arborized the whole striatum but also sent projections to the midbrain, thalamus, and HY (Figure 6B and C), and received afferent projections from the PFC, midbrain, and HY (Figure 8C). It is possible that hMOs bear a greater resemblance to the *de novo* SN A9 DA neural progenitors/precursors, and this intrinsic closeness in identity, together with a more homogeneous population with fewer contaminating cells, may endow hMOs with a higher capacity to establish bidirectional synaptic connections with the natural projection targets in the brain. It is noteworthy that the high level of arborization could also be attributed to the species difference in graft vs. host, as developmentally, human neurons need to travel a longer distance and therefore possess a greater migratory and arborizing abilities. Future studies employing non-human primate PD models would be informative to learn more about the safety and efficacy of hMOs.

## Materials and Methods

### Cells culture and generation of hMOs

hiPSCs were cultured on a feeder-free Matrigel in the E8-mTESR medium (STEMCELL^TM^, Vancouver, Canada; catalog # 05990). Induction of the hMOs was carried out according to a protocol previously described by Jo and Nolbrant S with minor modification 61, 65. Briefly, intact colonies of hiPSCs were dissociated into single cells using TrypLE^TM^ Express (Gibco, Waltham, MA, USA; 12563029), and 10,000 cells/well were plated low-cell-adhesion 96-well culture plates (Corning, Corning, NY, USA). The wells contained neuronal induction medium, which contained DMEM/F12^TM^ (Gibco, 10565-018): Neurobasal^TM^ (Gibco, 21103049) (1:1), 1:100 N2^TM^ supplement (Gibco, 17502048), 1:50 B27^TM^ without vitamin A (Gibco, 12587010), 1% minimum essential media-nonessential amino acid (Gibco, 11130051), and 0.1% β-mercaptoethanol (Invitrogen, Waltham, MA, USA; 31350010) supplemented with 10 μM SB431542 (Stemgent, Cambridge, MA, USA), 100 ng/mL Noggin (Peprotech, Rocky Hill, NJ, USA), 0.8 μM CHIR99021 (Selleck, S2924), SHH (300 ng/mL R&D system; 200ng/mL Peprotech) and 10 μM ROCK inhibitor Y27632 (Calbiochem, San Diego, CA, USA). ROCK inhibitor was added for the first 48 h and the neuronal induction medium was changed every 2 days. On day 9, the supplements were removed and replaced with 100 ng/mL FGF8 (Peprotech) for midbrain patterning. After 2 days, the media was completely removed, and 30 μL of reduced growth factor Matrigel (BD Biosciences, Franklin Lakes, NJ, USA; 356230) was added to each well. Then, the hMOs were cultured in media with 20 ng/mL brain-derived neurotrophic factor (BDNF) and 200 μM ascorbic acid (Sigma-Aldrich, St. Louis, MO, USA). To promote growth and differentiation, the hMOs were transferred to ultra-low-attachment 6-wellplates (Costar, Washington, DC, USA). The plates contained the final differentiation media with 20 ng/mL BDNF (Peprotech), 10 ng/mL glial cell line-derived neurotrophic factor (GDNF) (Peprotech), 200 μM ascorbic acid, 500 μM db-cAMP (Sigma-Aldrich), and 1 μM DAPT (Sigma-Aldrich). hMOs were cultured using an orbital shaker (set at 70 rpm). The medium was replaced every three days. The induction of the iPSC-derived DA neurons in the 2-D culture was carried out using the same growth factors as used for the induction of hMOs.

### PD model establishment and transplantation of hMOs

All animal experiments were conducted following the 3R principle and approved by the ethics committee of Xuanwu Hospital Capital Medical University. Eighty-six SCID mice (8-12 weeks old, Charles River, Beijing, China) were included in this study, of which 76 were used to establish a PD model, as previously reported 36. Briefly, 2 μL 6-OHDA (5 mg/mL in saline with 0.2% ascorbic acid) was directly injected into the right striatum (anterior-posterior [AP] = + 0.5 mm, lateral [L] = - 2.1 mm, vertical [V] = - 3.2 mm). Four weeks after 6-OHDA lesioning, 26 mice with apomorphine-induced rotations exceeding 100 turns over a 30-min period were selected for transplantation experiments. Ten of the 26 6-OHDA-lesioned mice were allocated to the vehicle control group, which only received intrastriatal injection of transplantation buffer. The remaining 16 PD mice were allocated to the hMOs transplantation group. Three time points were selected following hMOs transplantation, and the number of mice allocated to each time points was as follows: 6 weeks (n = 4, group A), 12 weeks (n = 8, group B) and 16 weeks (n = 4, group C). One of 16 engrafted PD mice died accidentally during the course of the study. The remaining 15 engrafted PD mice showed graft survival, and the number of mice at each time point was as follows: 6 weeks (n = 4), 12 weeks (n = 8) and 16 weeks (n = 3). Prior to transplantation, the organoids were digested and counted to assess the cell number in each organoid. Three replicates were performed for each organoid. Organoids corresponding to 4 × 10^5^ cells were included and cut into small pieces. Each piece of tissue should be able to pass through a 10 μl pipette tip before it can pass through the needle without causing a blockage. Transplantable organoids were resuspended in 4 μl transplantation buffer containing 0.5 μM Rock inhibitor, B27 without vitamin A, and 20 ng/mL BDNF, which were then injected into the right striatum ([AP] = + 0.5 mm, lateral [L] = -2.1 mm, vertical [V] = -3.0 mm) using a microinjector (HAMILTON, SYR 1701N, needle size 26s G; Figure S4, G, and H). The injection rate was 0.5 μl/min, and the needle was left in place for an additional 5 min before it was slowly retracted.

### Construction of Rabies helper vectors and injection of G-rabies virus to examine the host-to-graft connectivity

Tracing and control vectors were purchased from AddGene (IDs: 30195 and 30456, respectively, Watertown, MA, USA). Human iPSCs (passages 30-35) were transduced with the lentiviruses expressing tracing or control vectors. Successfully transduced cells were confirmed by puromycin selection and GFP expression. The titer for the control virus, tracing virus, and RV-ENVA-G-mCherry G-rabies (BrainVTA company, Wuhan, China) was 4 × 10^8^ TU/mL, 2 × 10^8^ TU/mL, and 2 × 10^8^ TU/mL, respectively. A working dilution of 0.1-0.5% (about 1 μL per mouse in this study) was used for *in vivo* injection.

### Behavioral Test

#### Drug-induced Rotation Test

Apomorphine (APO)-induced rotations were investigated before and at various time points following transplantation of hMOs. Five minutes after intradermal APO injection (10 mg/mL in saline, 0.5 mg/kg), rotation was recorded. The total net number (contralateral minus ipsilateral) of rotations during a 30-min period was used for analysis.

#### Open field test

The spontaneous movement was assessed by using an automated open field system to measure the average speed and total moving distance moved. Mice were habituated to the open field arena (25.4 cm × 25.4 cm) for 10 min prior to the test, followed by a 30-min observation period. Each animal was tested at 1 week prior to organoid engraftment and re-tested at 6 weeks and 12 weeks post-transplantation. Open-field measurements are presented in meters (m) for the total moving distance moved.

#### Rotarod Test

Passive motor coordination function was tested by using a Harvard Instruments accelerating rotarod. To achieve a steady performance, all animals were pre-trained for two days. On day 1, the mice were trained three times on a spinning rod that advanced from 2 to 20 revolutions per min (rpm) in 300 s. On day 2, the mice were trained on a rod that was accelerated at 300 s from 3 rpm to 30 rpm twice and 4 rpm to 40 rpm once. The test began on the third day by using a revolving rod that was advanced from 4 to 40 rpm in 300 s. The duration of the stay on the rod was measured. An average length of three repeated tests for each animal was used for data analysis. GraphPad (GraphPad Software Inc., San Diego, CA, USA) was used for statistical analysis. For multiple comparisons, a two-way analysis of variance (ANOVA) followed by Bonferroni's post-hoc test was used.

### Tissue processing and immunofluorescent staining

Mice were perfused with 50 mL of 0.9% saline and 100 mL of ice-cold 4% paraformaldehyde (PFA) in phosphate-buffered saline (PBS). The brains were removed and preserved in 4% PFA for another 2 h, followed by re-suspension in 20% sucrose (in 0.1 M PB) overnight and in 30% sucrose for dehydration. Twelve series of 40-μm-thick coronal slices were collected. For immunofluorescence and immunohistochemical staining, tissues were blocked in 3% donkey serum for 2 h at room temperature. Table S2 lists the information on primary antibodies used as well as the working dilution. A peroxidase-based reaction followed by diaminobenzidine (DAB) precipitation (Goat anti-rabbit, ZSGB-BIO, Beijing, China) or appropriate Alexa 488, Alexa 555, or Alexa 647-conjugated secondary antibodies (Jackson ImmunoResearch, West Grove, PA, USA) were used for visualization.

### Gene expression analyses

A RNeasy kit was used to extract total RNA from hMOs on days 0, 7, 15, and 25 (Qiagen, Valencia, CA, USA). Total RNA was reverse transcribed (Quantitech, Qiagen) for qRT-PCR analysis. ABI Prism (Applied Biosystems, Waltham, MA, USA) was used for the qRT-PCR analysis. GAPDH was used as the reference gene.

### HPLC analysis

Organoid culture medium was replaced by HBSS (Gibco, Waltham, MA, USA) (3 mL per well) at 37 ℃ for 30 min and subsequently replaced with 3 mL high potassium-HBSS medium (60 mM KCL and 82 mM NaCl) containing the DA uptake blocker Nomifensine (10 μM, Sigma, St. Louis, MO, USA) and the monoamine oxidase inhibitor Pargyline (50 μM, Sigma), and incubated for 45 min at 37 ℃. Then 180 μL of high potassium medium from each well was transferred to dark Eppendorf tubes containing 20 μL 1N perchloric acid (Merck, Rahway, NJ, USA) with antioxidants (0.33g/L Na2S2O5 and 0.083g/L EDTA) on ice and stored at -80 ℃ until analysis. HPLC assay was used to measure the content of neurotransmitters in 100 µL of the culture supernatants.

### MED64 Multi-Electrode Array and Whole Cell Patch Clamp Recording

A multi-electrode array device was used to record the electrophysiological activity of hMOs on day 60. The area of each planar microelectrode in the MED64 Probe center was 50 μm × 50 μm, and the spacing was 150 μm (P515A model). Whole-cell patch- clamp recordings were used to analyze the electrophysiological activity of the engrafted EGFP+ organoids. Whole-cell patch clamp recordings were performed on acute brain slices using ACSF bath solution (125 mM NaCl, 2.5 mM KCl, 2 mM CaCl2, 1.25 mM NaH2PO4, 1 mM MgSO4, 25 mM glucose, and 26 mM NaHCO3). The internal solution consisted of 143 mM KCl, 8 mM NaCl, 10 mM HEPES, 1 mM MgCl2, 2% biocytin, 2 mM Na-ATP, and 0.4 mM Na-GTP. EGFP+ cells were visualized by using a fluorescence microscope.

### Microscopy and quantifications

A Leica microscope was used to capture bright-field images, and a Leica DMI6000 confocal microscope was used to obtain fluorescence images. To assess the numbers of EN1-, FOXA2-, OTX2-, NURR1-, and TH-expressing cells among the total DAPI-labeled cells *in vitro*, at least 10 sampling areas from each of the three organoids were counted by using the ImageJ software (National Institutes of Health, Bethesda, MD, USA). The graft on the brain slices was delineated, and images were captured using confocal microscopy to visualize the TH+ and hNA+ signals. Image J was used to manually count single- or double- labeled cells. Data are presented as the ratio of TH+ cells to total hNA+ cells.

### Statistical analysis

All data were presented as mean ± standard error of mean, and statistical analysis was carried out by using GraphPad Prism 9. A t-test was used to analyze the proportion of hNA+ cells that co-expressed TH. For all behavioral test results, two-way ANOVA followed by Bonferroni's multiple comparisons was used. For qRT-PCR analysis, one-way ANOVA was used. Statistical significance was set at a* p* value is ≤ 0.05. Significant levels were representative as *p < 0.05, ** p < 0.01, *** *p* < 0.001, **** *p* < 0.0001.

## Supplementary Material

Supplementary figures and tables.

## Abbreviations

fVM: Fetal ventral mesencephalon
DA: dopaminergic
2-D: 2-dimentional
PD: Parkinson's disease
hiPSCs: human induced pluripotent stem cells
hMOs: hMOs
SNpc: substantia nigra pas compacta
DBS: deep brain stimulation
ESCs: embryonic stem cells
iNSCs: induced neural stem cells
6-OHDA: 6-hydroxydopamine
DIV: days in vitro
HPLC: high-performance liquid chromatography
FGF8: fibroblast growth factor 8
SHH: Sonic hedgehog
BDNF: brain-derived neurotrophic factor
GDNF: glial cell-derived neurotrophic factor
cAMP: dibutyryladenosine 3′,5′-cyclic monophosphate sodium salt
DAPT: 1-dimethylethyl ester
SOX2: sry-box transcription factor 2
TH: tyrosine hydroxylase
TUJ1: tubulin beta 3 class III
OTX2: orthodenticle homeobox 2
FOXA2: forkhead box A2
EN1: engrail-1
NURR1: nuclear receptor subfamily 4
GIRK2: G-protein-coupled inward rectifier potassium
DIV: Days in vitro
ap.: apical
bas.: basal
min: minutes
EGFP: enhanced green fluorescent protein
hNA: human cell nuclei
hNCAM: human neural cell adhesion molecule
MSN: medium spiny neurons
QC: quercetin
APO: apomorphine
A-P: antero-posterior
PFC: prefrontal cortex
fa/CC: corpus callosum
MO: somatomotor cortex
SS: somatosensory cortex
NAc: nucleus accumbens
Strd/ Strv: dorsolateral/ventral striatum
GP: globus pallidus
VPL: ventral posterolateral nucleus of the thalamus
mfb: medial forebrain bundle
HY: hypothalamus
TH: thalamus
MB: midbrain
MRN: midbrain reticular nucleus
fr: fasciculus retro-flexus
SPF: sub-parafascicular nucleus
CLA: claustrum
EP: endopiriform nucleus
SI: substantia innominate
OT: olfactory bulb
SNr/ SNc: reticular/ compact part of substantial nigra
VTA: ventral tegmental area
NAc: nucleus accumbens
Strd/ Strv: dorsolateral/ventral striatum
TH: thalamus
PAL: pallidum
CTB: Cholera toxin subunit B
PFC: prefrontal cortex
HY: hypothalamus
Puro: puromycin
fi: fimbria
LHA: lateral hypothalamus area
