# Supplementary material for: Human iPSC-derived midbrain organoids functionally integrate into striatum circuits and restore motor function in a mouse model of Parkinson's disease
Source: Theranostics. 2023 Apr 29;13(8):2673–92. doi: 10.7150/thno.80271 (PMC10196819; doi:10.7150/thno.80271)
Supplement: Supplementary file 1 — Supplementary figures and tables. [file thnov13p2673s1.pdf]

Supplementary Materials

Human iPSC-derived midbrain organoids functionally integrate into striatum circuits and restore motor function in a mouse model of Parkinson's disease

Running title: hiPSC-derived midbrain organoids to treat PD

Xin Zheng[1, 2, 3, †], Deqiang Han[1, 2, 3, †], Weihua Liu[1, 2, 3], Xueyao Wang[1, 2, 3], Na Pan[4], Yuping Wang[4], Zhiguo Chen[1, 2, 3, *]

1 Cell Therapy Center, Beijing Institute of Geriatrics, Xuanwu Hospital Capital Medical University, National Clinical Research Center for Geriatric Diseases, and Key Laboratory of Neurodegenerative Diseases, Ministry of Education, Beijing 100053, China

2 Center of Neural Injury and Repair, Beijing Institute for Brain Disorders, Beijing, 100069, China

3 Center of Parkinson's Disease, Beijing Institute for Brain Disorders, Beijing, 100069, China

4 The Department of Neurology, Xuanwu Hospital Capital Medical University, Beijing 100053, China.

† These authors contributed equally to this work.

* Author of correspondence: Zhiguo Chen (chenzhiguo@gmail.com)

List of Supplementary Information

Supplementary Figure 1

Supplementary Figure 2

Supplementary Figure 3

Supplementary Figure 4

Supplementary Figure 5

Supplementary Figure 6

Supplementary Table 1: Primer information

Supplementary Table 2: Antibody Information

Supplementary Figure 1

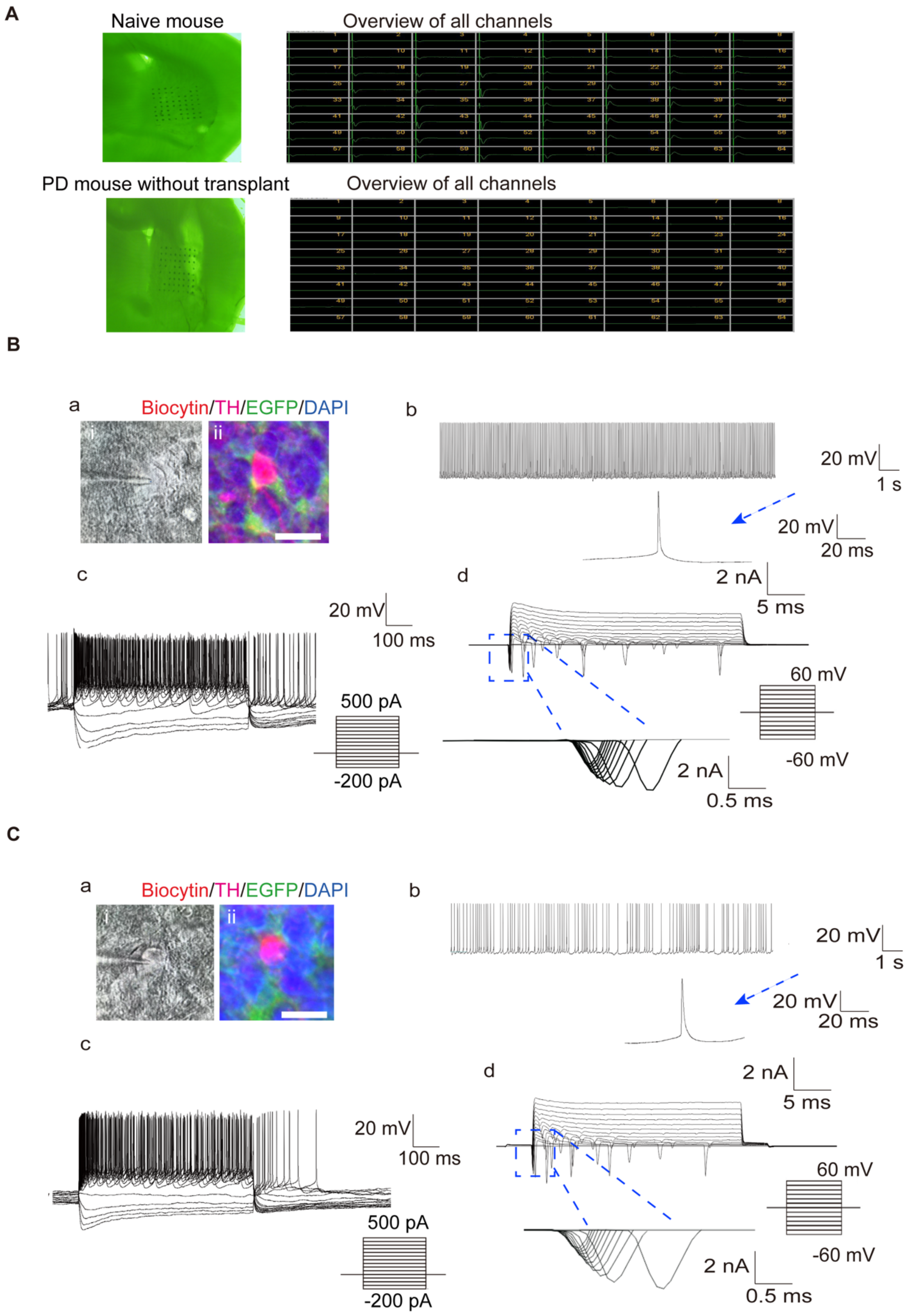

Figure S1. Electrophysiological properties of hMOs.

(A) The electrophysiological activity in naïve mice and 6-OHDA lesioned mice was

assessed via MED64 multi-electrode array system. **(B, C)** By using whole cell-patch clamp to identify the electrophysiological activity of grafted cells. (a) Phase contrast image of a patched neuron. Scale bars, 50 μm. (b) Regular spontaneous action potential (AP) activity of a transplanted cell. (c) APs evoked by step-current injections. (d) Representative voltage-dependent Na+ and K+ currents patched on a grafted neuron. Respective traces of inward Na+ in the blue box were expanded.

Supplementary Figure 2

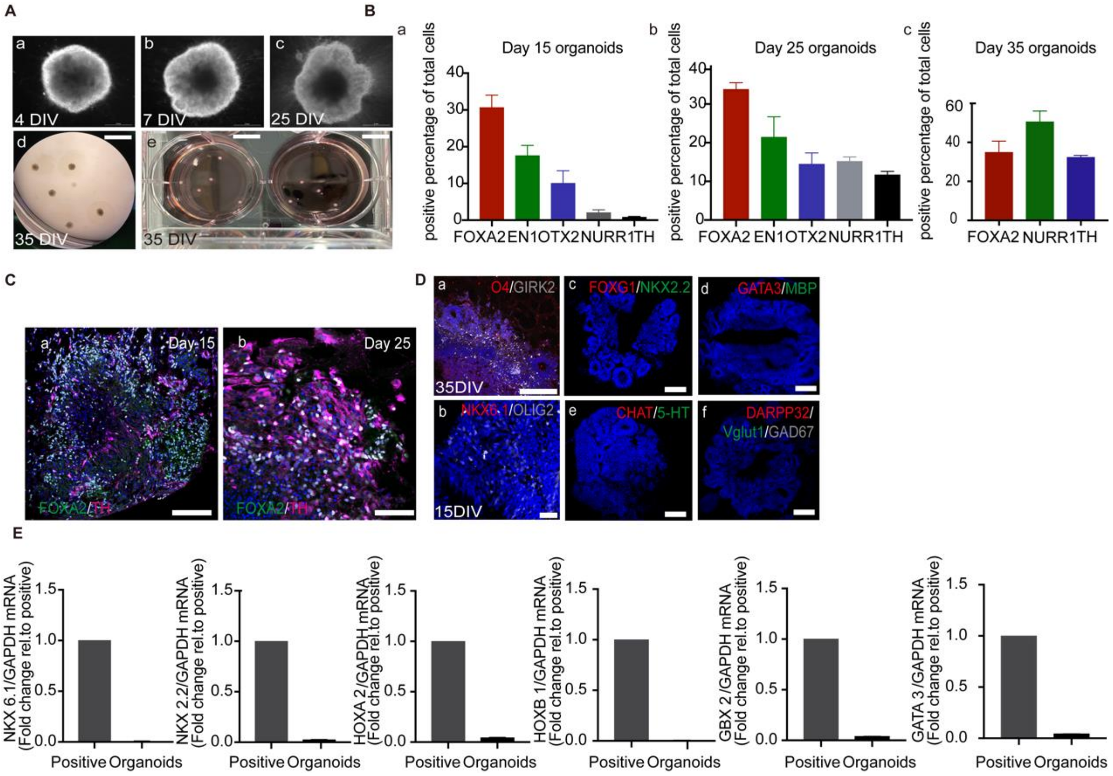

Figure S2. Cellular composition within human induced pluripotent stem cell (hiPSC)-derived 3-D midbrain organoids (hMOs).

(A) The morphology of organoids at different time points; images a-c taken by DIC, while image d and e were taken by a stereomicroscope and a camera, respectively. Scale bars, 2 mm. **(B)** Proportions of cells positive for individual dopaminergic neuron-related markers at days 15, 25 and day 35 ($n = 10$). **(C)** Representative images of 15-, and 25-day organoids. **(D)** Examination of non-DA cells. Few O4 + cells were detected on day 35 organoids (a) and few OLIG2 + cells detected on day 15 organoids (b). No cells positive for markers related to forebrain (FOXG1, c), hindbrain (NKX6.1, NKX2.2, GATA3) and other neuronal subtypes (including CHAT, 5-HT, DARPP32, Vglut1) were observed by immunofluorescence staining of day 15 organoids (e, f). Scale bars, 250 μm. **(E)** Expression of NKX 6.1, NKX 2.2, HOXA2, HOXB1, GBX2,

and GATA3 gene in Day 15 organoids confirmed the absence of serotonin progenitor cells.

Supplementary Figure 3

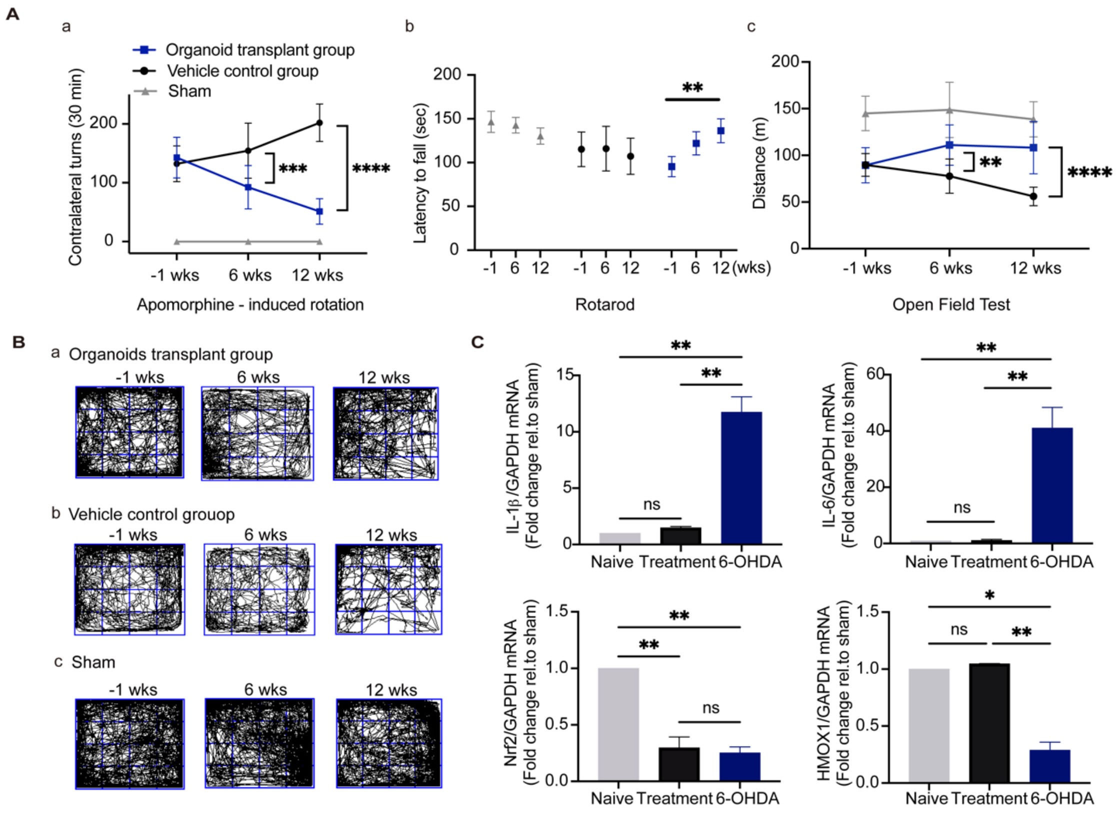

Figure S3. Behavioral assessment.

(A) Results of behavioral tests including apomorphine-induced rotation ($n = 8$) (i), rotarod (ii) and open field tests (iii). **(B)** Representative mouse moving traces in open field experiment. **(C)** Expression of proinflammatory and anti-inflammatory cytokines. Three repeated tests for each sample were performed.

Supplementary Figure 4

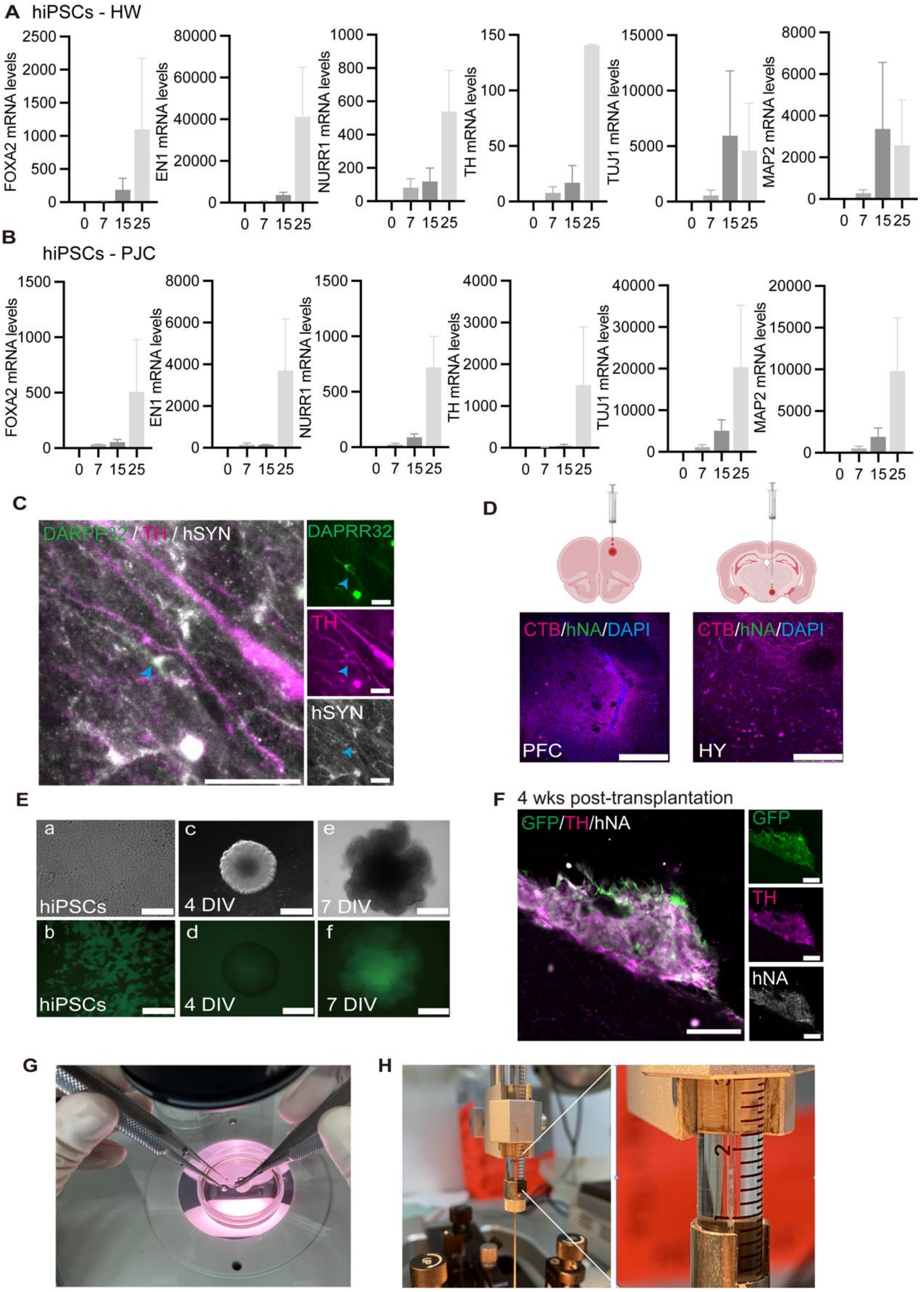

Figure S4. Transplantation of organoids.

Two different cell lines hiPSCs-HW **(A)** and hiPSCs-PJC **(B)** were used for evaluation.

Representative RT–PCR results at different differentiation time points. Results were presented as fold changes compared to undifferentiated hiPSCs at day 0. **(C)** Triple staining for TH, DARPP32 and hSYN confirmed the synaptic connection (arrowheads) between dopaminergic (DA) and medial spiny neuron (MSN). Scale bars, 50 μm; **(D)** The CTB deposits were clearly separated from the graft itself; CTB and hNA were double stained and the results showed that CTB signal was not detected within the hNA+ grafted cells. Scale bars, 250 μm. **(E)** hiPSCs, and days 4 and day 7 organoids expressing rabies glycoprotein (RVG) and EGFP. a, c and e, DIC images; b, d and f, live cell images captured using fluorescence microscopy. Scale bars, 1 mm; **(F)** Four weeks post-transplantation, maturated DA neurons with GFP expression were observed at the engraftment site. Scale bars, 50 μm. **(G)** The organoids were physically cut into small pieces. Each piece of tissue should be able to pass through a 10 μL pipette tip before it can pass through the needle without causing a blockage. **(H)** Transplantable organoids were resuspended in 4 μL transplantation buffer and then injected into the right striatum by using a microinjector.

Supplementary Figure 5

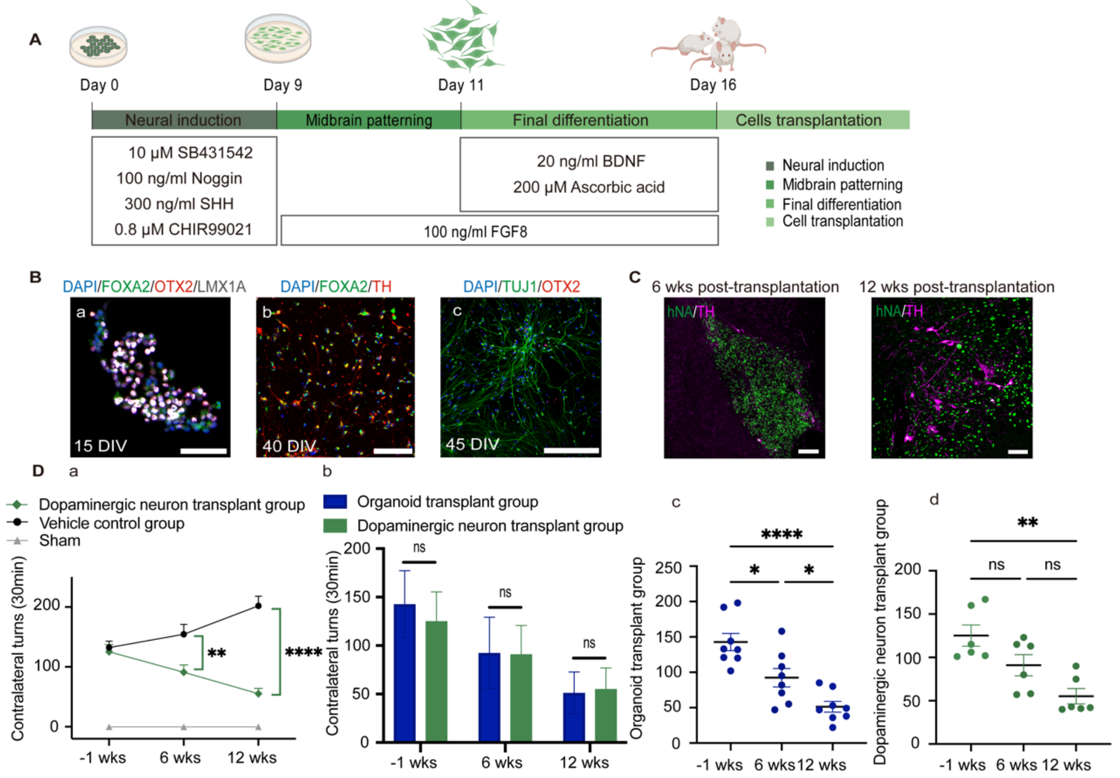

Figure S5. iPSC-derived DA precursors are cultured in a 2-D manner.

(A) Schematic representation of the differentiation procedure from hiPSCs to transplantable DA neurons. **(B)** Immunofluorescent staining for characterization of hiPSC-derived DA neurons (*n* = *10*). Scale bars: a, c, 250 *μ*m; b, 100 *μ*m. **(C)** Representative confocal images of TH+ cells among the engrafted cells at two time points following transplantation (*n* = *6*). Scale bars, 100 *μ*m. **(D)** Results of apomorphine-induced rotation (*n* = *6*).

Supplementary Figure 6

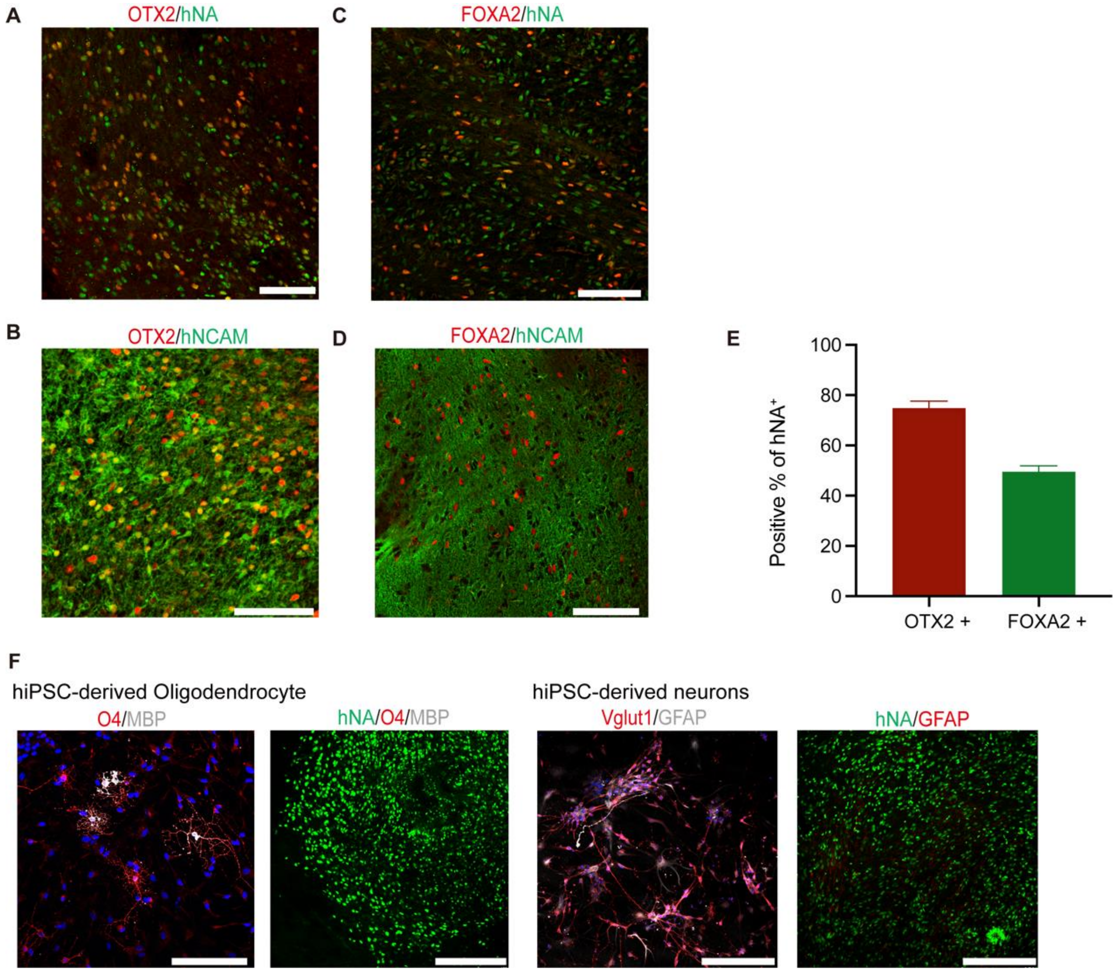

Figure S6. Characterization of the engrafted cells *in vivo*.

(A-D) Co-expression of OTX2+/hNA+ and FOXA2+/hNA+ in the grafts following implantation. Scale bars, 100 μm. **(E)** The proportions of cells positive for individual markers among the engrafted cells (74.83% for OTX2+ and 49.50% for FOXA2+). **(F)** iPSC-derived- astrocytes, oligodendrocytes and other types of neurons (glutamatergic neurons, for example) were used as positive controls, and these glial cells or neuronal subtypes were not detected within the grafted deposit *(n = 8)*. Scale bars, 250 μm.

Supplementary Table 1: Primer information

Gene	Entrez ID	Forward	Reverse
GAPDH (Human)	2597	AAGAAGGTGGTGAAGCAGG	AGGTGGAGGAGTGGGTGTCG
NESTIN	10763	GTAGCTCCCAGAGAGGGGAA	CTCTAGAGGGCCAGGGACTT
SOX2	6657	AGTCTCCAAGCGACGAAAAA	TTTCACGTTTGCAACTGTCC
FOXA2	3170	GGGGTAGTGCATCACCTGTT	CCGTTCTCCATCAACAACCT
EN1	2019	TCTCGCTGTCTCTCCCTCTC	CGTGGCTTACTCCCCATTTA
OTX2	5015	CCAGACATCTTCATGCGAGAG	GGCAGGTCTCACTTTGTTTTG
NURR1	4929	GCTGGACTCCCCATTGCTTT	CGGAGCTGTATTCTCCCGAA
TH	7054	TGTCTGAGGAGCCTGAGATTCG	GCTTGTCCTTGGCGTCACTG
TUJ1	10381	GACCCCAGCGGCAACTAC GTG	ACGTACTTGTGAGAAGAGGCCTCGT
NKX6.1	4825	ACACGAGACCCACTTTTTCCG	GCCCCGCCAAGTATTTTGTT
NKX2.2	4821	AAACCATGTCACGCGCTCA	GGCGTTGTACTGCATGTGCT
HOXA2	3199	CGTCGCTCGCTGAGTGCCTG	TGTCGAGTGTGAAAGCGTCGAGG
HOXB1	3211	GAGCTTTGCACCGGCCTAT	CTTCATCCAGTCGAAGGTCCG
GBX2	2637	CTCGCTGCTCGCCTTCTC	GCCAGTCAGATTGTCATCCG
GATA3	2625	GCCCCTCATTAAGCCCAAG	TTGTGGTGGTCTGACAGTTCG
GAPDH (Mouse)	14433	GGTCGGAGTCAACGGATTTGGTCG	CCTCCGACGCCTGCTTCACCAC
IL-1β	16176	GAAATGCCACCTTTTGACAGTG	TGGATGCTCTCATCAGGACAG
IL-6	16193	TACCACTTCACAAGTCGGAGGC	CTGCAAGTGCATCATCGTTGTTC
Nrf2	18024	TTCTTTCAGCAGCATCCTCTCCAC	ACAGCCTTCAATAGTCCCGTCCAG
HMOX1	3162	GCACCGGCCGGATGGAGCGTCC	CGTCTCGGGTCACCTGGCCCTTCTG

Supplementary Table 2: Antibody information

Primary antibody	Species	Vendor	Catalog#	Dilution	Assay
NESTIN	Mouse	BD bioscience	611658	1:500	IF
NESTIN	Mouse	Millipore	MAB353	1:500	IF
SOX2	Rabbit	Invitrogen	48-1400	1:200	IF
KI67	Rabbit	Millipore	AB9260	1:500	IF
OCT4	goat	Abcam	AB27985	1:200	IF
FOXA2	Rabbit	Millipore	07-633	1:200-500	IF
FOXA2	Goat	Santa cruz	Sc-6554	1:200-500	IF
EN1	Mouse	Developmental Studies Hybridoma Bank (DSHB)	4G11	1:20-100	IF
EN1	Rabbit	Abgent	AP7278A	1:250	IF
OTX2(biotinylated)	Goat	R&D Systems	AF1979	1:50-200	IF
NURR1	Rabbit	Santa cruz	sc-991	1:200	IF
NURR1	Mouse	Abcam	Ab41917	1:200	IF
NURR1	Goat	R&D Systems	AF2156	1:100	IF
TH	Rabbit	Millipore	AB152	1:200	IF, IHC
TH	Sheep	Millipore	AB1542	1:200	IF
TH	Mouse	ImmunoStar	22941	1:500	IF
TUJ1	Mouse	Millipore	MAB1637	1:500	IF
Girk2	Rabbit	Alomone Labs	APC-006	1:100	IF
Girk2	Goat	Abcam	Ab65069	1:100	IF
Human-specific Synaptophysin 1	Goat	R&D Systems	AF5555	1:100	IF
Synaptophysin	Mouse	Synaptoc systems	101011	1:500	IF
Synaptophysin	Rabbit	Abcam	AB32127	1:200	IF
Serotonin transporter (5-HT)	Rabbit	Abcam	Ab254358	1:200	IF
GFP	Rabbit	Invitrogen	A-11122	1:500	IF
GFP	Goat	Genscript	A01703	1:200	IF
hNA	Mouse	Millipore	MAB1281	1:500	IF
hNCAM	Mouse	Santa cruz	Sc-106	1:100-200	IF
DARPP32	Rabbit	Abcam	Ab40801	1:200	IF
CTB	Goat	List labs	703	1:100	IF
mCherry	Rat	Invitrogen	M11217	1:200	IF
FOXG1	Rabbit	Abcam	Ab18259	1:200	IF
CHAT	Goat	Millipore	AB144P	1:200	IF
Calbindin	Rabbit	Abcam	AB108404	1:200	IF
Vgluta1	Goat	Abcam	Ab110139	1:200	IF
GAD67	Chicken	Abcam	Ab75712	1:200	IF
GAD67	Mouse	Millipore	MAB5406	1:200	IF

GABA	Rabbit	Millipore	A2052	1:200	IF
Olig2	Rabbit	Millipore	AB9610	1:500	IF
O4	Mouse	R&D	MAB1326	1:200	IF
MBP	Rat	Abcam	Ab7349	1:200	IF
NKX2.2	Mouse	DSHB	74.5A5-s	1:200	IF
NKX6.1	Mouse	DSHB	F55A12-s	1:200	IF
GATA3	Mouse	R&D	MAB6330	1:200	IF
GFAP	Rabbit	ZSGB-BIO	ZA-0529	1:500	IF
